# COVID-19 vaccination dynamics in the US: coverage velocity and carrying capacity based on socio-demographic vulnerability indices in California's pediatric population

**DOI:** 10.3389/fpubh.2023.1148200

**Published:** 2023-05-09

**Authors:** Alexander A. Bruckhaus, Azrin Khan, Trevor A. Pickering, Aidin Abedi, Sana Salehi, Dominique Duncan

**Affiliations:** ^1^Laboratory of Neuro Imaging, USC Stevens Neuroimaging and Informatics Institute, Keck School of Medicine of USC, University of Southern California, Los Angeles, CA, United States; ^2^Department of Population and Public Health Sciences, Keck School of Medicine of USC, University of Southern California, Los Angeles, CA, United States; ^3^USC Neurorestoration Center, Keck School of Medicine, University of Southern California, Los Angeles, CA, United States; ^4^Department of Neurological Surgery, Keck School of Medicine, University of Southern California, Los Angeles, CA, United States; ^5^Rancho Research Institute, Rancho Los Amigos National Rehabilitation Center, Downey, CA, United States

**Keywords:** COVID-19, COVID-19 vaccines, epidemiology, social vulnerability, public health

## Abstract

**Introduction:**

COVID-19 vaccine inequities have been widespread across California, the United States, and globally. As COVID-19 vaccine inequities have not been fully understood in the youth population, it is vital to determine possible factors that drive inequities to enable actionable change that promotes vaccine equity among vulnerable minor populations.

**Methods:**

The present study used the social vulnerability index (SVI) and daily vaccination numbers within the age groups of 12–17, 5–11, and under 5 years old across all 58 California counties to model the growth velocity and the anticipated maximum proportion of population vaccinated.

**Results:**

Overall, highly vulnerable counties, when compared to low and moderately vulnerable counties, experienced a lower vaccination rate in the 12–17 and 5–11 year-old age groups. For age groups 5–11 and under 5 years old, highly vulnerable counties are expected to achieve a lower overall total proportion of residents vaccinated. In highly vulnerable counties in terms of socioeconomic status and household composition and disability, the 12–17 and 5–11 year-old age groups experienced lower vaccination rates. Additionally, in the 12–17 age group, high vulnerability counties are expected to achieve a higher proportion of residents vaccinated compared to less vulnerable counterparts.

**Discussion:**

These findings elucidate shortcomings in vaccine uptake in certain pediatric populations across California and may help guide health policies and future allocation of vaccines, with special emphasis placed on vulnerable populations, especially with respect to socioeconomic status and household composition and disability.

## Introduction

**Figure 1:**
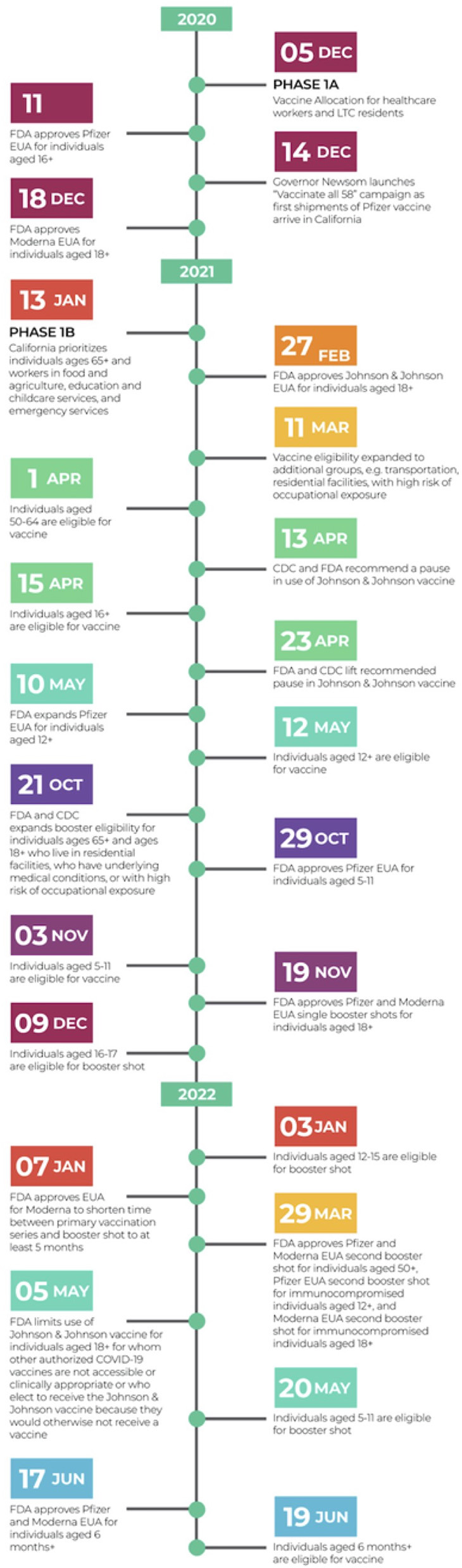
Chronological summary of major COVID-19 vaccination related events and policies nationwide and state-wide (California). Relative to pediatric population: On April 15, 2021, all Californians aged 16+ years became eligible for the COVID-19 vaccine (4). On May 12, 2021, all Californians aged 12+ years became eligible for the COVID-19 vaccine (2). On October 29, 2021, FDA approved Pfizer EUA for individuals aged 5–11 years old (5). On November 3, 2021, all Californians aged 5–11 years old became eligible for the COVID-19 vaccine (6). On December 9, 2021, Californians aged 16–17 years old became eligible for the COVID-19 booster shot (7). On January 3, 2022, Californians aged 12–15 years old became eligible for the COVID-19 booster shot (8). On January 7, 2023, FDA approved EUA for Moderna to shorten time between primary vaccination series and booster shot to at least 5 months (9). On Mach 29, 2022 FDA approved Pfizer and Moderna EUA second booster shot for individuals aged 50+, Pfizer EUA second booster shot for immunocompromised individuals aged 12+, and Moderna EUA second booster shot for immunocompromised individuals aged 18+ (10). On May 20, 2022, Californians aged 5–11 years old became eligible for the COVID-19 booster shot (11). On June 17, 2022, the FDA approved Pfizer and Moderna EUA for individuals aged 6 months and older (12). On June 19, 2022, Californians aged 6 months and older became eligible for the COVID-19 vaccine (3). CDC, Centers for Disease Control and Prevention; EUA, Emergency Use Authorization; FDA, Food and Drug Administration.

Coronavirus disease 2019 (COVID-19) vaccination administration inequities have been widespread both across the United States (U.S.) as well as globally. While these inequities exist on a national and a global scale, they have also been found to exist within the state of California (1). However, the inequities within the minor population have not yet been fully understood. With children aged 12–15 years old only gaining access to the COVID-19 vaccine in May of 2021 and children under the age of 5 only gaining access in June of 2022 (see Figure 1), COVID-19 vaccine administration inequities must be investigated in order for there to be actionable and informed changes to improve such inequities before they fully manifest themselves (2, 3).

**Figure 2:**
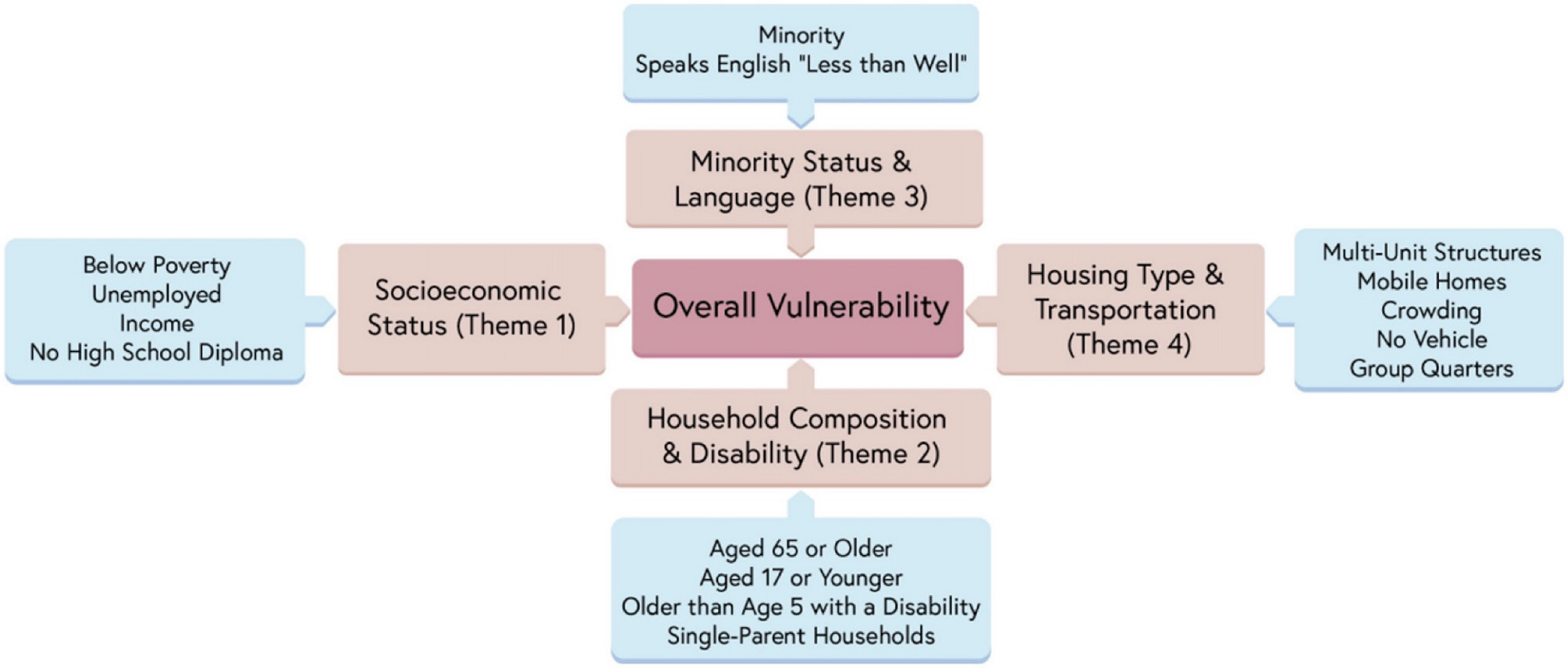
Social Vulnerability Index (SVI), underlying themes, and components. Reprinted by permission from Springer Nature (1).

To quantify levels of vulnerability across California, the present study uses a metric called the Social Vulnerability Index (SVI), which is composed of four underlying themes (and an all-encompassing overall measure), which are (1) socioeconomic status, (2) household composition and disability, (3) minority status and language, and (4) housing type and transportation, as detailed in Figure 2 (1).

Extending prior research where SVI was used as a metric to estimate coverage velocity and the maximum anticipated proportion of vaccinated individuals in California counties (1), we once again use California counties as our population of interest. Because of its stature as the highest populated state in the U.S. (13), along with its vast diversity and high minority population (40.2% Hispanic or Latinx, 35.2% White, 15.9% Asian, and 6.5% Black as of 2021 estimates) (14), analysis of the Californian population provides opportunities to investigate minority and highly heterogeneous populations.

While the CDC has used other indexes such as the US COVID-19 Community Vulnerability Index (CCVI) and the Pandemic Vulnerability Index (PVI) during the COVID-19 pandemic to conduct public health research, the SVI has consistently and recently been used as an index to quantify levels of vulnerability in the context of vaccine uptake, among the pediatric and adult population (15–19), rendering it a valuable and reliable index. Although other indexes like the CCVI and PVI build off of the SVI and provide utility to communities, the SVI has been used longer and has been cited as a reliable measure to provide local and state public health officials vital information to effectively allocate resources and take proper action to ameliorate vaccine inequities (20). Previous studies have successfully used the SVI to measure disparities in COVID-19 health outcomes, revealing inequities in vaccine uptake across the U.S. (1, 15, 19, 21). One study showed that vaccine disparities were most highly attributed to socioeconomic status (SVI Theme 1) and household composition and disability (SVI Theme 2) status (21). Of interest to our study, however, is how the relationship between SVI themes and vaccine administration unfolds in the pediatric population (age groups 12–17, 5–11, and under 5). Kim et al. (15) utilized the SVI to identify vaccine administration inequities borne by children aged 5–11 years old, and found the largest disparities in high vulnerability areas, which were attributed to racial, ethnic, and socioeconomic disparities. Although not a measure incorporated into the SVI, vaccine hesitancy is also a factor that cannot be discounted and has been shown to be intertwined with vaccine uptake and SVI (22). In particular, vaccine hesitancy among parents or primary caregivers has been shown to be associated with lower median household income (23). It has also been found that highly vulnerable populations tend to experience higher vaccine access over time, with the gap between low and highly vulnerable counties shrinking (1, 15). While we focus our analysis on all age groups of California children under 18 years old, we note that California has only recently begun to offer COVID-19 vaccines to children under 5 years of age. It is vital to monitor these age-specific trends to uncover patterns and address areas of concern in vaccine administration, as no known studies have been conducted using SVI for this age group at the time of the study. At the time of data collection for this study (July 9, 2022), we estimate that 3.73% (83,545 out of 2,239,667) of individuals in California aged under 5 have received the COVID-19 vaccine (at least one dose), 40.4% (1,417,100 out of 3,507,810) of individuals aged 5–11 have received the COVID-19 vaccine (at least one dose), and 72.96% (2,334,546 out of 3,199,686) of individuals aged 12–17 have received the COVID-19 vaccine (at least one dose). To date (March 25, 2023), we estimate that 12.95% (290,060 out of 2,239,667) of individuals aged under 5 have received the COVID-19 vaccine (at least one dose), 43.33% (1,520,209 out of 3,507,810) individuals aged 5–11 have received the COVID-19 vaccine (at least one dose), and 74.27% (2,376,641 out of 3,199,686) of individuals aged 12–17 have received the COVID-19 vaccine (at least one dose). All numerators were gathered from, while denominators were the sum of estimated populations from all 58 counties for each age group (24). Thus, it is vital to study trends within uptake among these populations to ensure equitable distribution of COVID-19 vaccines.

Therefore, in a similar fashion to Bruckhaus et al. (1), the present study aimed to determine whether the longitudinal trends in vaccination rates across California counties differ by SVI in the pediatric population. To achieve this goal, we modeled the growth rate in vaccination coverage and the anticipated maximum proportion of vaccinated individuals in relation to county-level rankings in overall SVI and its four underlying themes (1). To obtain a more granular understanding of the trends among the minor population, we separated children into three distinct age groups: Under five years old, 5–11 years old, and 12–17 years old.

## Materials and methods

### Data acquisition

The process of data acquisition for the current study is similar to that of our previous study (1). Namely, we acquired 2018 Social Vulnerability Index (SVI) data from the Agency for Toxic Substances and Disease Registry (ATSDR)'s Geospatial Research, Analysis & Services Program (GRASP) (25), while we acquired vaccination data (initial vaccination and booster data) from the California Health & Human Services Agency (CHHS) (24). Data were acquired for three different age groups, including 12–17 years of age, 5–11 years of age, and under 5 years of age.

Counties were classified as either low, moderate, or high vulnerability based on ranking all 58 California counties by vulnerability rating and splitting them into tertiles. Rankings were assigned to SVI Theme 1 (Socioeconomic Status), Theme 2 (Household Composition and Disability), Theme 3 (Minority Status and Language), Theme 4 (Housing Type & Transportation), and overall SVI. For a full breakdown of each theme, (see Figure 2). For more information on the SVI, visit the SVI documentation (25).

### Data curation

Data curation was similar to our previous paper (1), except for the population segments studied and the time frame in which their data were analyzed. That is, the three age groups analyzed (12–17 years of age, 5–11 years of age, and under 5 years of age) had different initial vaccine eligibility dates, dependent on when the COVID-19 vaccine became available to these populations. In the 12–17 age group, the start date for individuals with at least 1 dose was May 12, 2021 while the start date for booster shots was January 3, 2022, which corresponds to about 6 months after the initial second dose eligibility. In the 5–11 age group, the start date for individuals with at least 1 dose was November 3, 2021, while the start date for booster shots was May 20, 2022, which corresponds to about 6 months after initial eligibility. In the under-five age group, the start date for individuals with at least 1 dose was June 19, 2022, while authorization for a booster shot for this age group only recently became available in December of 2022. R software version 4.1.1 and the tidyverse package were used to compile and clean the data. The proportion of residents with at least one vaccine dose contains a numerator of the cumulative count of residents with at least one dose of any authorized vaccine, while the denominator is the estimated total population of each age group in each county (1, 24). Proportion of residents with a booster shot was computed similarly.

## Modeling strategy and statistical analysis

Initial plots of the longitudinal curves for each county of cumulative proportion of children with initial vaccination dose, and proportion of children with a booster, were created to determine the shape of the longitudinal growth curves. Because denominator data was only available for the entire 12–17 year age group, we treated the beginning of eligibility for this group as May 12, 2021, even though children age 16–17 had been eligible since April 15, 2021. Thus, the longitudinal curves for the 12–17 year age group begin at proportions >0.

The curves for proportion with initial vaccination dose and booster dose appeared to follow that of an exponential saturation model, in which the instantaneous change in the proportion of population vaccinated at time *t* is given as a function of the per capita growth rate *r* and the asymptotic maximum proportion *K*:


$$\begin{array}{l} \frac{dp}{dt}=r\left(K-p_{t}\right) \end{array}$$

And the proportion of individuals vaccinated at time t is given by:


$$\begin{array}{l} p_{t}=K-\left(K-p_{0}\right)e^{-rt} \end{array}$$

We used a non-linear least-squares model fitting approach using the nls function in R (v4.1.1) to fit these equations. Of interest was whether the parameters r (coverage velocity) and K (anticipated maximum proportion of population vaccinated) (and for the 12–17 year group, *P*_0_) differed based on SVI group. To accomplish this, we included dummy variable interaction terms into the model to allow for differences in these parameters based on SVI category. Let i_1_ and i_2_ be indicators of a county having “moderate” and “high” SVI, respectively. The model equation can then be written as:


$$\begin{array}{c} p_{t}=\left(K+dK_{1}i_{1}+dK_{2}i_{2}\right)\\ -\left(\left(K+dK_{1}i_{1}+dK_{2}i_{2}\right)-p_{0}\right)e^{-\left(r+dr_{1}i_{1}+dr_{2}i_{2}\right)t} \end{array}$$

Where dK_1_ and dK_2_ represent the difference in asymptotic maxima, and dr_1_ and dr_2_ represent the difference in growth rate, for moderate and high SVI counties, respectively, compared to low SVI counties. SVI-specific estimated growth rates and asymptotic maxima parameters were computed using the lincom function in the biostat3 package. The performance of the exponential saturation model was compared to other similar models, such as logistic growth, non-linear splines, and polynomial terms, using *R*^2^.

To examine “snapshots” of how vaccination disparities by SVI status at the beginning of eligibility persisted through time, we fit a negative binomial regression model to examine the rate ratios of proportion vaccinated in each of the SVI groups. Time points of snapshots varied depending on the age group studied. In these models, the cumulative number of individuals vaccinated was used as the outcome, with an offset of the natural log of county population size. These models included an interaction term between SVI category and date to test whether the proportional differences in vaccination rates persisted through the study period.

We found that certain counties had data inconsistencies and were excluded from our analysis. Imperial county had reported values that gave a proportion of individuals with initial vaccination dose that exceeded 1.0. Alpine county did not have vaccination information for individuals aged 12–17 years. Alpine, Lassen, Modoc, Sierra, and Trinity counties had poor data for children under age 5 years.

## Results

### Initial vaccination

#### Model parameter values

**Figure 3:**
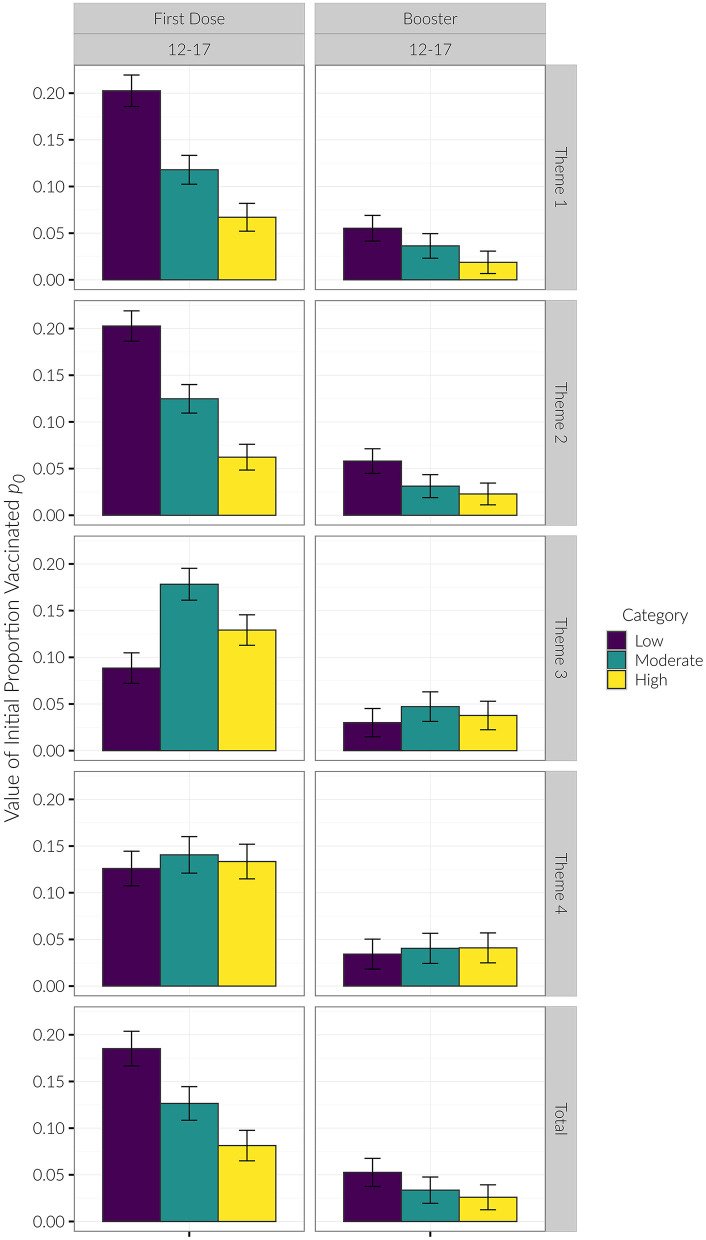
*p*_0_ values. *P*_0_ was only different for Themes 1 & 2 for the 12–17 age group First Dose. Lower SVI had higher initial proportion vaccinated. In Theme 3, Moderate had the highest proportion vaccinated, followed by High, followed by Low SVI. The reason *p*_0_ was different for this category was because the 12–17 age group had eligibility for 16–17 year olds prior to the initial eligibility date modeled.

**Figure 4:**
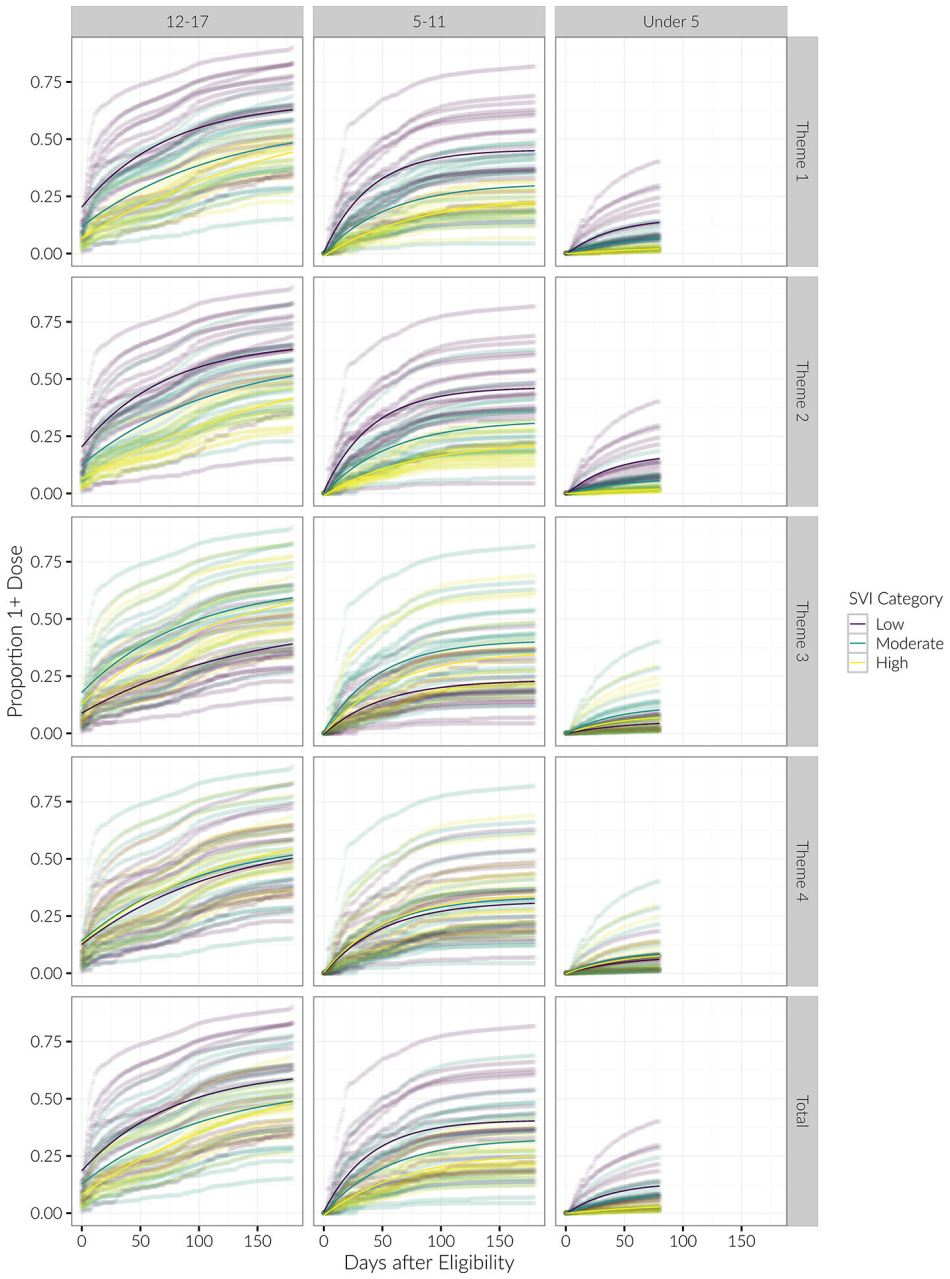
Longitudinal trends of cumulative proportion of individuals with at least one vaccine dose, beginning at date of eligibility. Curves for each county are shown, with average trends in bold. Results are stratified by age category and SVI theme.

Examining initial vaccination rates, the *R*^2^ for the exponential saturation model was the highest out of all the models across the three age groups. Model performance was the best when stratifying counties by SVI Theme 1 (*R*^2^ = 0.53, 0.54, 0.40 for the 12–17, 5–11, and Under 5 age groups, respectively) and Theme 2 (R^2^ = 0.57, 0.58, 0.51). For these themes, in the 12–17 age group, low vulnerability counties had the highest initial proportion of vaccinated children, followed by moderate vulnerability, then high vulnerability. For Theme 3 in the 12–17 age group, moderate vulnerability counties had the highest initial proportion of vaccinated children, followed by high vulnerability, then low vulnerability counties (see Figures 3, 4). The initial proportion vaccinated was trivial in the 5–11 and Under 5 age groups.

**Figure 5:**
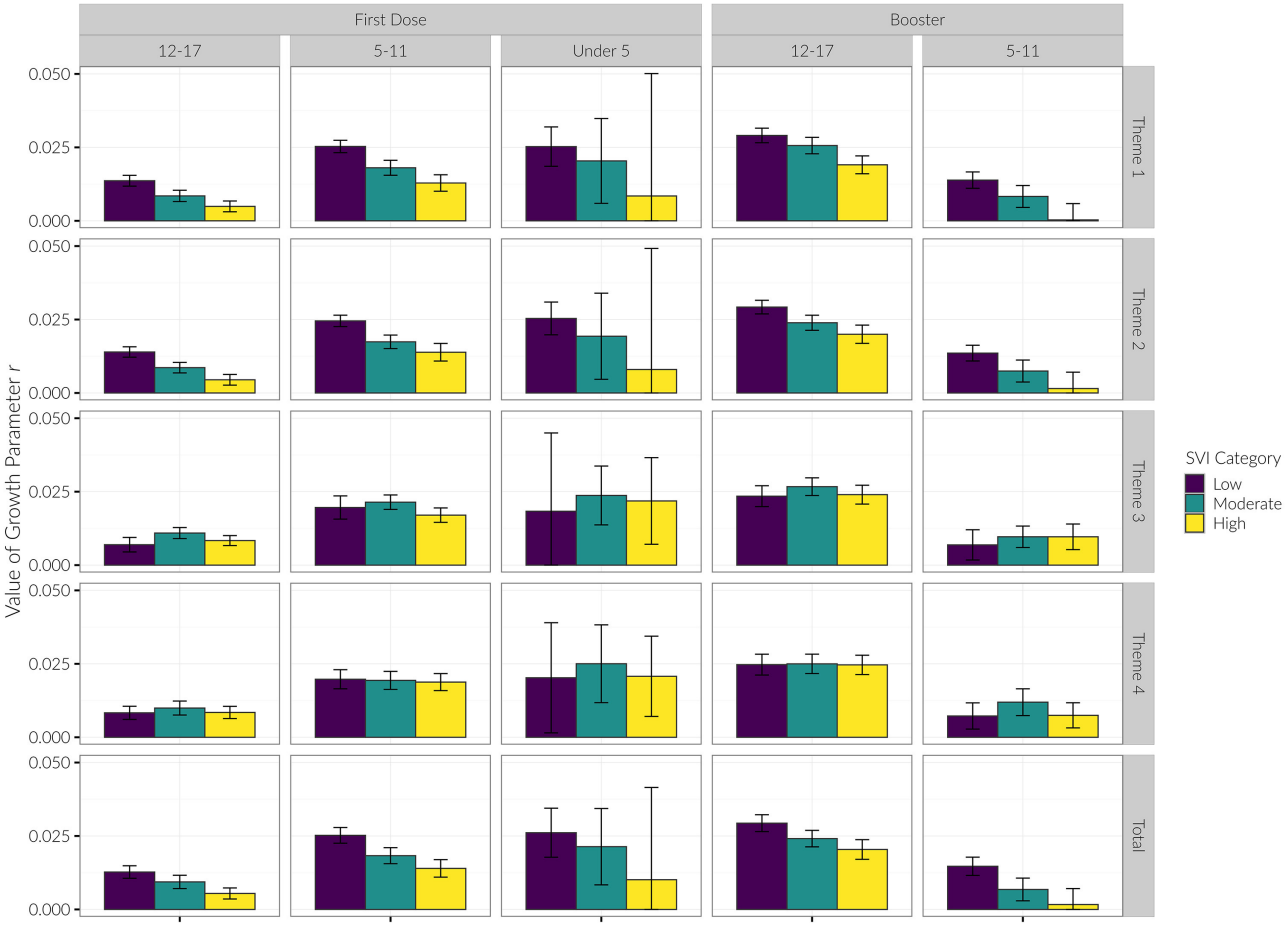
Growth parameter (*r*) of each age group for initial vaccine dose and booster shots by SVI category.

We saw similar trends regarding the growth parameter *r* (see Table 1; Figure 5). That is, for Themes 1 and 2, low vulnerability counties had the largest rate of growth during the study period, followed by moderate, then high vulnerability counties. This effect was seen in the 12–17 and 5–11 age groups, but there was not a statistically significant difference in *r* for the Under 5 age group. For Theme 3, the moderate vulnerability group had the highest growth rate in the 12–17 and 5–11 age groups.

**Table 1:** Parameter estimates of *n, r*, and *K* for all age groups within overall SVI and Themes 1–4 for at least one vaccine dose.

**SVI**	**Total**	**Theme 1**	**Theme 2**	**Theme 3**	**Theme 4**
**12–17**					
***n***					
Low	0.185 (0.167, 0.204)	0.203 (0.186, 0.219)	0.203 (0.186, 0.219)	0.088 (0.072, 0.105)	0.126 (0.107, 0.144)
Moderate	0.126 (0.108, 0.144)	0.118 (0.102, 0.133)	0.125 (0.109, 0.14)	0.178 (0.161, 0.195)	0.141 (0.121, 0.16)
High	0.081 (0.065, 0.098)	0.067 (0.052, 0.082)	0.062 (0.048, 0.076)	0.129 (0.113, 0.146)	0.133 (0.115, 0.152)
***r***					
Low	0.013 (0.011, 0.015)	0.014 (0.012, 0.015)	0.014 (0.012, 0.016)	0.007 (0.004, 0.009)	0.008 (0.006, 0.011)
Moderate	0.009 (0.007, 0.012)	0.008 (0.007, 0.01)	0.009 (0.007, 0.01)	0.011 (0.009, 0.013)	0.01 (0.008, 0.012)
High	0.005 (0.004, 0.007)	0.005 (0.003, 0.007)	0.004 (0.003, 0.006)	0.008 (0.007, 0.01)	0.008 (0.006, 0.011)
***K***					
Low	0.631 (0.603, 0.66)	0.668 (0.646, 0.691)	0.667 (0.646, 0.688)	0.512 (0.427, 0.598)	0.611 (0.544, 0.678)
Moderate	0.572 (0.521, 0.623)	0.586 (0.532, 0.639)	0.619 (0.568, 0.671)	0.66 (0.624, 0.696)	0.591 (0.542, 0.64)
High	0.724 (0.584, 0.865)	0.708 (0.55, 0.867)	0.702 (0.525, 0.879)	0.696 (0.638, 0.754)	0.655 (0.591, 0.72)
**5–11**					
***r***					
Low	0.025 (0.023, 0.028)	0.025 (0.023, 0.027)	0.025 (0.023, 0.026)	0.02 (0.016, 0.024)	0.02 (0.017, 0.023)
Moderate	0.018 (0.016, 0.021)	0.018 (0.015, 0.021)	0.017 (0.015, 0.02)	0.021 (0.019, 0.024)	0.019 (0.016, 0.022)
High	0.014 (0.011, 0.017)	0.013 (0.01, 0.016)	0.014 (0.011, 0.017)	0.017 (0.015, 0.019)	0.019 (0.016, 0.022)
***K***					
Low	0.407 (0.398, 0.417)	0.454 (0.446, 0.462)	0.464 (0.456, 0.472)	0.234 (0.221, 0.247)	0.315 (0.3, 0.329)
Moderate	0.328 (0.314, 0.342)	0.308 (0.295, 0.32)	0.32 (0.307, 0.333)	0.407 (0.396, 0.419)	0.337 (0.322, 0.351)
High	0.274 (0.252, 0.295)	0.251 (0.229, 0.273)	0.225 (0.206, 0.243)	0.361 (0.345, 0.377)	0.349 (0.334, 0.364)
**Under 5**					
***r***					
Low	0.026 (0.018, 0.034)	0.025 (0.019, 0.032)	0.025 (0.02, 0.031)	0.018 (−0.008, 0.045)	0.02 (0.002, 0.039)
Moderate	0.021 (0.008, 0.034)	0.02 (0.006, 0.035)	0.019 (0.005, 0.034)	0.024 (0.014, 0.034)	0.025 (0.012, 0.038)
High	0.01 (−0.021, 0.042)	0.008 (−0.033, 0.05)	0.008 (−0.033, 0.049)	0.022 (0.007, 0.037)	0.021 (0.007, 0.034)
***K***					
Low	0.136 (0.118, 0.155)	0.157 (0.139, 0.176)	0.176 (0.159, 0.193)	0.057 (0.013, 0.1)	0.076 (0.041, 0.111)
Moderate	0.098 (0.069, 0.127)	0.078 (0.051, 0.106)	0.071 (0.043, 0.099)	0.121 (0.098, 0.144)	0.099 (0.076, 0.123)
High	0.05 (−0.059, 0.16)	0.042 (−0.111, 0.195)	0.039 (−0.111, 0.189)	0.084 (0.057, 0.111)	0.094 (0.063, 0.125)

SVI, social vulnerability index; *r*, growth parameter (coverage velocity); *K*, carrying capacity (estimated maximum proportion of individuals vaccinated).

#### Snapshots analysis

**Figure 6:**
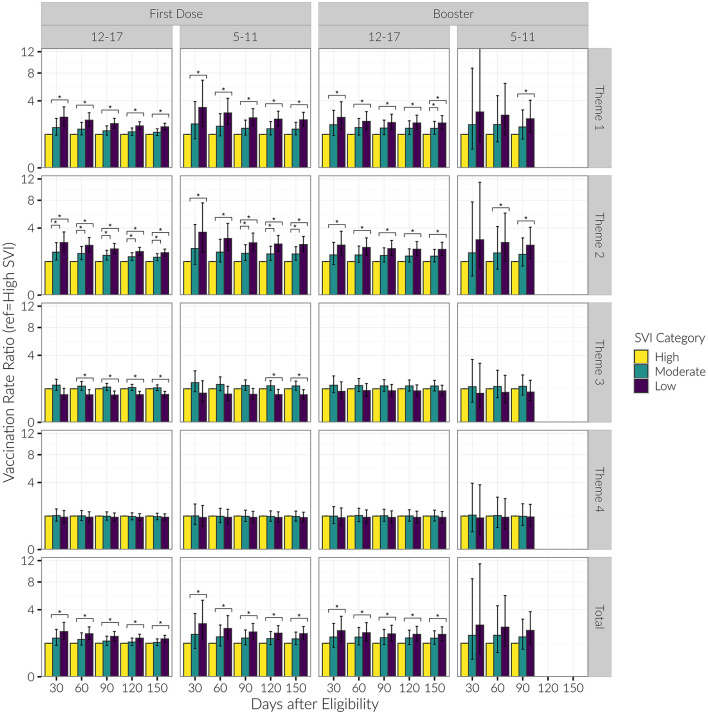
Vaccination rate ratios for low and moderate SVI vs. high SVI counties, by date and SVI category, for age groups of 12–17 and 5–11 years old. Presented are rate ratios with 95% confidence intervals. *Indicates a difference in the vaccination rate between SVI categories (baseline group = “High SVI”).

We did not compute the snapshots analysis for the Under 5 age group due to unstable estimates caused by low vaccination rates. For Theme 1 in both the 12–17 and 5–11 age groups, the low SVI group consistently had higher rates of vaccination compared to the high SVI group through the entire period of observation. Additionally for Theme 2 in the 12–17 age group, moderate SVI counties also had consistently higher initial vaccination rates compared to high SVI counties. This trend was also observed in the 5–11 age group, but only beginning at 90 days after eligibility. For Theme 3, low SVI counties had lower initial vaccination rates in comparison to high SVI counties beginning 60 days after eligibility for the 12–17 age group and beginning 120 days after eligibility for the 5–11 age group. We observed no differentiation in vaccination rates among SVI categories for Theme 4 (see Supplementary Table 1; Figure 6).

### Booster vaccination

#### Model parameter values

**Figure 7:**
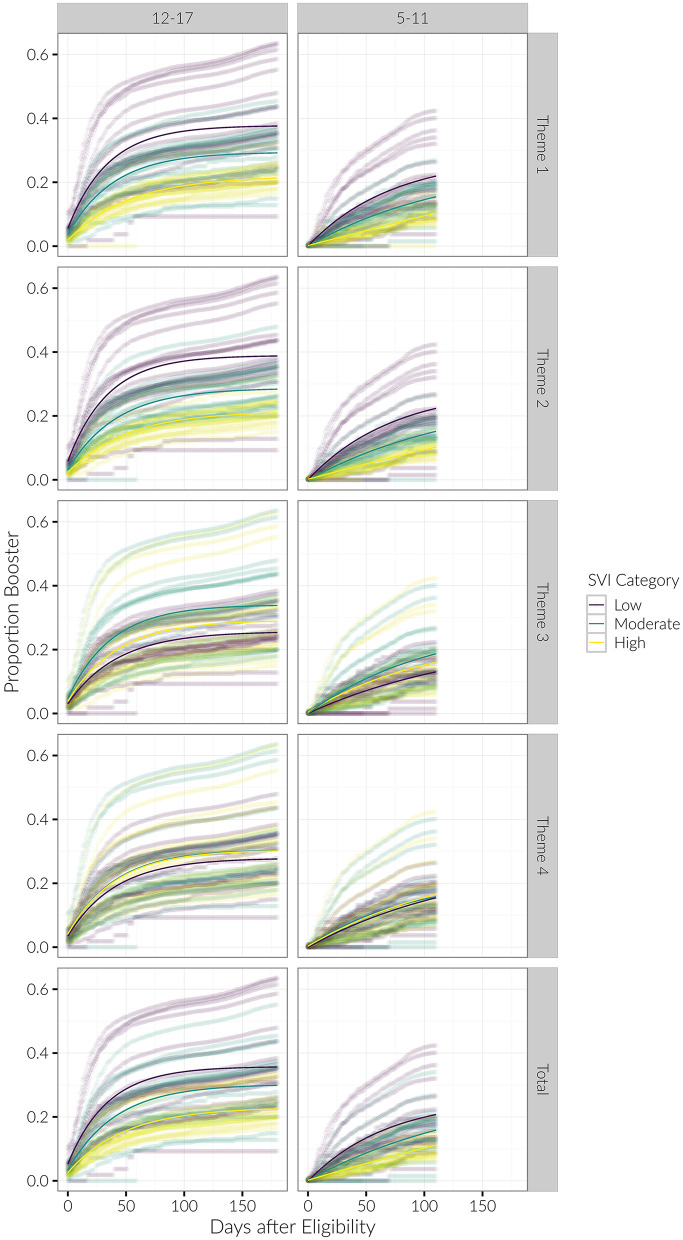
Longitudinal trends of cumulative proportion of individuals with booster, beginning at date of eligibility. Curves for each county are shown, with average trends in bold. Results are stratified by age category and SVI theme.

We generally observed the same trends with the booster as we did with the initial vaccination dose. Regarding booster rates, the *R*^2^ for the exponential saturation model was again highest out of all the models across the three age groups. Model performance was the best when stratified by SVI Theme 1 (*R*^2^ = 0.52, 0.53 for the 12–17 and 5–11 age groups, respectively) and Theme 2 (*R*^2^ = 0.56, 0.54). Similar to the initial vaccine dose, there was a decreasing initial proportion of children vaccinated with increasing vulnerability category in Themes 1 and 2 in the 12–17 age group (see Figures 4, 7). Again, the initial proportion vaccinated was trivial in the 5–11 age group. Model parameters for the booster dose were not stable in the Under 5 age group due to the sparsity of data and are not reported here.

The growth parameter *r* was less strongly differentiated among SVI categories for the booster dose than it was for the initial vaccination dose. However, for Themes 1 and 2 we again observed that the growth parameter *r* consistently decreased with increasing SVI vulnerability in both the 12–17 and 5–11 age groups (see Table 2; Figure 5).

**Table 2:** Parameter estimates of *n, r*, and *K* for age groups 12–17 and 5–11 years old within overall SVI and Themes 1–4 for booster shot.

**SVI**	**Total**	**Theme 1**	**Theme 2**	**Theme 3**	**Theme 4**
**12–17**					
***n***					
Low	0.053 (0.038, 0.068)	0.055 (0.042, 0.069)	0.058 (0.045, 0.071)	0.03 (0.015, 0.045)	0.034 (0.018, 0.05)
Moderate	0.034 (0.02, 0.048)	0.036 (0.023, 0.05)	0.031 (0.019, 0.044)	0.047 (0.031, 0.063)	0.04 (0.024, 0.057)
High	0.026 (0.013, 0.039)	0.019 (0.007, 0.031)	0.023 (0.011, 0.034)	0.038 (0.022, 0.053)	0.041 (0.025, 0.057)
***r***					
Low	0.029 (0.026, 0.032)	0.029 (0.027, 0.032)	0.029 (0.027, 0.032)	0.023 (0.02, 0.027)	0.025 (0.021, 0.028)
Moderate	0.024 (0.021, 0.027)	0.026 (0.023, 0.028)	0.024 (0.021, 0.026)	0.027 (0.024, 0.03)	0.025 (0.022, 0.028)
High	0.02 (0.017, 0.024)	0.019 (0.016, 0.022)	0.02 (0.017, 0.023)	0.024 (0.021, 0.027)	0.025 (0.021, 0.028)
***K***					
Low	0.358 (0.352, 0.364)	0.378 (0.372, 0.383)	0.389 (0.384, 0.395)	0.257 (0.249, 0.265)	0.279 (0.271, 0.287)
Moderate	0.303 (0.295, 0.31)	0.294 (0.288, 0.301)	0.287 (0.281, 0.294)	0.341 (0.334, 0.348)	0.306 (0.298, 0.314)
High	0.231 (0.222, 0.239)	0.219 (0.21, 0.228)	0.215 (0.207, 0.223)	0.292 (0.284, 0.3)	0.305 (0.297, 0.313)
**5–11**					
***r***					
Low	0.015 (0.012, 0.018)	0.014 (0.011, 0.017)	0.014 (0.011, 0.016)	0.007 (0.002, 0.012)	0.007 (0.003, 0.012)
Moderate	0.007 (0.003, 0.011)	0.008 (0.005, 0.012)	0.007 (0.004, 0.011)	0.01 (0.006, 0.013)	0.012 (0.007, 0.017)
High	0.002 (−0.004, 0.007)	0 (−0.005, 0.006)	0.002 (−0.004, 0.007)	0.01 (0.005, 0.014)	0.007 (0.003, 0.012)
***K***					
Low	0.259 (0.232, 0.286)	0.28 (0.252, 0.309)	0.289 (0.259, 0.318)	0.246 (0.117, 0.374)	0.282 (0.162, 0.401)
Moderate	0.301 (0.181, 0.42)	0.259 (0.183, 0.336)	0.272 (0.179, 0.364)	0.286 (0.22, 0.352)	0.216 (0.17, 0.261)
High	0.664 (−1.325, 2.653)	3.096 (−52.314, 58.507)	0.66 (−1.583, 2.903)	0.243 (0.177, 0.31)	0.293 (0.18, 0.405)

SVI, social vulnerability index; *r*, growth parameter (coverage velocity); *K*, carrying capacity (estimated maximum proportion of individuals vaccinated).

#### Snapshots analysis

For Theme 1 and 2 in the 12–17 age category, the low SVI group consistently had higher rates of vaccination compared to the high SVI group through the entire period of observation. For the 5–11 age category, the low SVI group had higher booster vaccination rates beginning at 90 days for Theme 1, and at 60 days for Theme 2. Booster vaccination doses did not vary significantly among county SVI groups when stratified by Themes 3 or 4 (see Supplementary Table 1; Figure 6).

### Theme 2 analysis

After finding that Theme 2 most strongly differentiated county vaccination rates, we conducted additional exploratory analyses. We stratified counties based on the two components of Theme 2: Disability and Single-Parent Household. We found that disability most strongly differentiated rate of growth for the initial vaccination dose (*R*^2^ = 0.68) compared to the single-parent household variable (*R*^2^ = 0.35). That is, low SVI counties had the highest initial proportion vaccinated and the steepest rate of growth in comparison to moderate and high SVI counties. The effect was similar when examining the single-parent household variable, but the parameter estimates were not as strongly differentiated among SVI groups for this measure. See Supplementary Figures 1–3 and Supplementary Tables 2–4.

## Discussion

The present study elucidated vaccine administration inequities across low, moderate, and highly vulnerable counties across the pediatric population (under age five, ages 5–11, and ages 12–17) in California. Overall SVI and SVI Themes 1–4, along with longitudinal vaccination data across all 58 California counties were analyzed to estimate the disparities in the maximum anticipated proportion of vaccinated individuals based on the current coverage velocity. Through our analysis, we demonstrated that, across the age groups of 12–17 and 5–11 years old, counties deemed highly vulnerable in regard to overall SVI, Theme 1 (socioeconomic status), and Theme 2 (household composition & disability) experienced lower coverage velocities when compared to moderate or low vulnerable counties. We also observed that in the age groups of under age 5 and 5–11 years of age, highly vulnerable counties with respect to overall SVI, Themes 1 and 2 have among the lowest anticipated maximum proportion of vaccinated individuals compared to low and moderately vulnerable counties. Interestingly, the opposite was seen for the age group of 12–17 year-olds, with Theme 1 and Theme 2 showing the highest anticipated maximum proportion of individuals vaccinated, relative to low and moderately vulnerable counties.

In terms of access to vaccination facilities, information regarding the location of vaccination for the population studied is not available in the dataset. However, individuals may receive a COVID-19 vaccination in a myriad of locations and facilities. According to County of Los Angeles, Public Health, common facilities in which children can receive a COVID-19 vaccine include clinics, hospitals, pharmacies, pop-up locations, drive-thru locations, schools, and public transit stations (26). However, the availability and presence of these facilities varies widely by county, so certain counties may have administered COVID-19 vaccinations more or less frequently among different facilities.

The present study demonstrates the importance and utility of the SVI and its application to elucidating COVID-19 vaccine disparities in communities. It has been previously applied to the California adult population to identify disparities in the initial COVID-19 vaccine rollout (1). By using the SVI, communities can better understand weaknesses and strengths in their communities so public health officials and emergency response planners can more efficiently allocate vital resources to their communities (25). The CDC and other peer-reviewed studies suggest that by utilizing the insights provided by the SVI to identify counties with low vaccination rates, state and local governments can prioritize their efforts and allocate funds toward regions that are most in need of the COVID-19 vaccine (15, 17–19). Within these studies, some specific actions that can be taken to address inequities revealed by the SVI are community outreach programs, transmitting culturally relevant information to communities, teaming up with trusted messengers to provide accurate and timely information to communities, and ensuring accurate and timely supply of COVID-19 vaccines to communities (15, 17–19). Furthermore, insights provided by the SVI can assist local officials and policymakers to help eliminate vaccine barriers by providing streamlined vaccine offers such as community vaccine clinics and mobile clinics, which could help eliminate the fear of taking time off work for many parents who wish to vaccinate their children. An example of how identification of vulnerable populations has helped ameliorate vaccine inequities comes from California's campaign to provide support to the hardest hit communities in March of 2021. Although the Healthy Places Index was used to identify vulnerability in these decisions, actions such as providing extra allotment of vaccines, public education programs, and culturally relevant information to vulnerable communities demonstrate that identifying vulnerable communities can lead to informed and impactful decision making (27).

Akin to several prior studies which analyzed disparities among vaccination uptake, our findings bolster the existing link between COVID-19 vaccine uptake and socioeconomic status and household composition & disability (21–23). Our study extends the findings of Barry et al., 2021 who reported disparities based on SVI Themes 1 and 2 (21), albeit their sample included adults too. Moreover, we found that these inequities were generally consistent through all three age groups in the present study. Although socioeconomic status and household composition & disability may broadly explain patterns of inequities, parental hesitancy can also not be discounted as an underlying contributor to the inequities we bear witness to. Yet, the two factors of socioeconomic status and parental hesitancy may be closely related, as one study found an inverse relationship between income and childhood vaccine hesitancy (23), while other studies also point to parental vaccine hesitancy as a barrier to childhood COVID-19 vaccination (15, 22). In what follows, we attempt to explore the underlying factors that may explain the inequities in vaccine administration for children in California, while identifying parallels between the present study and our previous study which used SVI to identify COVID-19 vaccine allocation disparities in the California adult population.

While Theme 3 and Theme 4 did not contain the level of stark disparities as we observed in Theme 1 and 2, as evidenced by the K and r parameters and the snapshot analysis, we see that Themes 3 and 4 continue the trend of our previous study of COVID-19 vaccine inequity in California (1). That is, in Theme 3 of our previous study, we observed that communities deemed highly vulnerable began with the lowest vaccination coverage velocity, but soon surpassed low vulnerable counties about 100 days after initial rollout, when California assisted these communities with public education and funding toward the COVID-19 vaccine. This change corresponds to about March of 2021. In the present study, we observe that highly vulnerable counties in all age groups (with the initial date of analysis for ages 12–17 on May 12, 2021) for Theme 3 have a higher coverage velocity than low vulnerable counties. In other words, our previous study indicates that policies and strategies to increase vaccine uptake among certain vulnerable subgroups may have been effective by the time vaccine rollout began for children. Therefore, our findings regarding Theme 3 may represent a continuation of the trends that we previously observed among the adult population (1), highlighting the importance of resource allocation and public education, which may ultimately improve vaccine uptake. Similarities in trends are also observed in Theme 4, although there were insignificant differences in vaccine uptake among vulnerability statuses.

### Theme 1: socioeconomic status

Among the four SVI themes analyzed in the present study, we now explore differences in vaccination uptake relative to Theme 1 (socioeconomic status). Among age groups of 12–17 years old and 5–11 years old, highly vulnerable counties experienced a significantly lower vaccination rate than low and moderately vulnerable counties. As for the estimated maximum proportion of individuals vaccinated, we see a similar pattern for age groups under 5 years of age and 5–11 years of age, as highly vulnerable Theme 1 counties see a lower estimated maximum proportion of individuals vaccinated compared to low and moderately vulnerable counties. Interestingly, however, we observed that for the age group of 12–17, highly vulnerable counties relative to Theme 1 are estimated to achieve a higher proportion of vaccinated individuals.

Prior studies have suggested that lower income parents or guardians may face additional challenges in vaccinating their children. Although, overall, a significant proportion of children aged 5–11 in the U.S. live within 5 miles of a pediatric vaccine provider (92%) (15), factors within the SVI as well as parental hesitancy, may affect parents, guardians, and children's decisions to get vaccinated. Despite relative proximity to vaccine providers, parents may be less likely to take time away from work to get their child vaccinated (22), or still, more likely to face transportation challenges, which become exacerbated with a paucity of pediatric or family medicine facilities in these areas (22). Even when the COVID-19 vaccine is swiftly available for most communities in California, the dissemination of reliable and timely information is crucial for improving vaccine coverage in these areas, particularly by trusted community leaders (15). Trusted, accessible, and thorough information regarding the safety and efficacy of vaccines are especially vital in lower-income areas, as studies have shown an association between household income and vaccine hesitancy (23).

It has been observed in several studies, as mentioned above, that parental hesitancy may lead to lower vaccination rates in children. Expanding on this relationship, recent studies found that vaccination hesitancy was higher among low income, non-college graduate adults, and publicly insured parents (22, 23, 28, 29). According to prior studies, the majority of vaccine-hesitant adults are more concerned about vaccine safety than vaccine effectiveness, less knowledgeable about vaccines, and generally less disturbed about the health risks related to COVID-19 (28, 29). Lack of resources and misinformation about vaccines have greatly impacted vaccine hesitancy. A qualitative study on a highly vulnerable county in California reported that participants feared differential vaccine treatment (well-resourced communities presumed to fare better) and felt allocation ambiguity (uncertainties including requirements and insurance policies) and cost uncertainty (30). This historical fear of discrimination and mistrust in the healthcare system creates barriers to adequate health services for individuals in highly vulnerable counties, especially among lower income areas.

As for the estimated maximum proportion of individuals vaccinated in the 12–17 year old age group, previous literature appears to be aligned with our finding that highly vulnerable counties are on pace to achieve higher vaccination numbers than low and moderate vulnerable counties in Themes 1 and 2. A study conducted on vaccination coverage among adolescents from July to October 2021 reported a large increase in vaccination uptake when parents had an income of <$35,000 (31). While this study focuses on the U.S. population at large, we nonetheless observe a similar trend in the present study as our snapshot analysis reveals that the disparity between highly vulnerable counties and moderate and low vulnerable counties pertaining to socioeconomic status becomes ameliorated throughout July to October 2021. While COVID-19 vaccines are not mandated in schools yet (32), a study reported that parents of 12–17 year olds who were encouraged and informed by their child's school about COVID-19 vaccinations were more likely to get their children vaccinated (this response increased since July 2021) than those whose child's school did not encourage vaccination (33). This study also reported that parents without college degrees, compared to college graduates, are more likely to be concerned about their children getting seriously ill from COVID-19. Overall, schools' outreach efforts and parents' concerns about COVID-19 may help explain our findings pertaining to the higher anticipated proportion of individuals vaccinated in the 12–17 years old age group.

### Theme 2: household composition and disability

Another prominent source of COVID-19 vaccine uptake inequity lies within Theme 2 (household composition & disability). Because Theme 2 consists of variables such as individuals aged 65 or older and 17 or younger, and the present study focuses on children under age 18, we further broke down this analysis for all age groups (except for the snapshot analysis of under age 5) to include parameter estimates for the variables of “Older than Age 5 with a Disability” and “Single-Parent Households” (see Supplementary material). Within this analysis, we discovered that coverage velocity among highly vulnerable counties was far lower than low and moderately vulnerable counties for the variables of disability and single-parent households, with disability showing slightly lower coverage velocity than single-parent households in the 12–17 year old age group. Our snapshot analysis reveals starker disparities over time between high and low vulnerable counties for the disability variable compared to the single-parent variable.

In terms of the disability variable, the SVI metric incorporates the number of civilians in a household with a disability. Therefore, it may be the case that either a child or a parent has a disability. Not surprisingly, highly vulnerable counties within this classification have a lower coverage velocity, as previous studies have shown significant barriers for vaccination in this population. A study by the CDC reported that adults with disabilities may experience challenges such as difficulty scheduling an appointment online, not knowing where to get vaccinated, going to vaccination sites, and vaccination sites not being open at convenient times (34). While the present study focuses on COVID-19 vaccine uptake in children, some of these difficulties directly affect children, while others may carry over to adults who are disabled, intending to vaccinate their children. If it were the case that minors had disabilities, actionable change to ensure caregivers are able to receive informative, trustworthy information about the COVID-19 vaccine should be considered (15). For example, trusted leaders or governments can distribute informative flyers and increase public education surrounding the COVID-19 vaccine and its associated safety details pertaining to the disabled population. With the proper safety and efficacy knowledge, caregivers can become more informed with their decision to vaccinate their children with disabilities. The concern that highly vulnerable counties—relative to the disability variable—have a lower coverage velocity is amplified as people with disabilities may be at a higher risk of infection and severe illness due to underlying medical conditions (35).

While the disparity between high and low and moderately vulnerable counties in terms of the single-parent household variable is not as pronounced as that of disability status, we offer possible explanations to the disparities observed within the single-parent household variable. While there is a paucity of literature surrounding the relationship between vaccination uptake in children and single-parent households, a 2022 study revealed that vaccine hesitancy was associated with single-parent households (36), which may help explain the disparities we observe between high and low and moderately vulnerable counties.

### Limitations

The present study does not come without limitations. Firstly, while individuals aged 16+ were eligible for the COVID-19 vaccine beginning on April 15, 2021, children aged 12+ became eligible on May 12, 2021. Because the California COVID-19 vaccine data only had an age group of 12–17 years old (for individuals aged 12+), we set the initial eligibility date to May 12, 2021 to account for this limitation. Furthermore, in rare cases, data showed that individuals in certain age groups were being vaccinated before their eligibility date, which may be attributed to special populations such as the immunocompromised. Due to the nature of the analysis, it is unlikely that this limitation influenced the final findings. Second, within the 12–17 year old age group, certain counties had data inconsistencies and therefore were excluded. This included (1) Imperial county, which had reported numbers that provided a proportion of individuals with initial vaccination dose that exceeded 1.0, and (2) Alpine county, for which vaccination information for individuals aged 12–17 years was not reported. Third, within the 5–11 year old age group, Imperial county had data inconsistencies and was therefore excluded. Fourth, while the under age 5 population only recently became eligible (June 2022) for the COVID-19 vaccine, the data in this segment is not as robust as the other age groups. However, we do see similar trends in this age group (inequities regarding Theme 1 and Theme 2) relative to the other age groups. Furthermore, we excluded several counties in this age group due to data quality issues (Imperial and Alpine) or missing values (Lassen, Modoc, Sierra, and Trinity). Finally, although vaccinations were reported for each county, the specific location or facility in which individuals were vaccinated were not reported. These data may have provided more detailed insights into inequities brought upon by availability and access to vaccine locations.

## Conclusion

The present study shines light on the scope of COVID-19 vaccine inequity in the pediatric population within California. We demonstrated the strengths and weaknesses of the current state of vaccine uptake while also drawing parallels to historic trends, current events, and future directions. By addressing COVID-19 vaccine inequities and their potential sources, interventions can more thoroughly be planned and implemented to improve public health across California, the U.S., and the world.

## Data availability statement

All data used in the present study is publicly available and can be found on the internet at any time. California COVID-19 vaccination data can be found at: https://data.ca.gov/dataset/covid-19-vaccine-progress-dashboard-data/resource/766edceb-8d41-4086-9856-4fb2538cec5f?inner_span=True. SVI data and documentation can be found at: https://www.atsdr.cdc.gov/placeandhealth/svi/data_documentation_download.html.

## Author contributions

AB: conceptualization, data curation, writing—original draft, and writing—review and editing. AK and AA: conceptualization, writing—original draft, and writing—review and editing. TP: data curation, formal analysis, investigation, methodology, software, validation, visualization, writing—original draft, and writing—review and editing. SS: conceptualization and writing—review and editing. DD: conceptualization, funding acquisition, project administration, supervision, and writing—review and editing. All authors contributed to the article and approved the submitted version.

## References

[1] Bruckhaus AA Abedi A Salehi S Pickering TA Zhang Y Martinez A. COVID-19 vaccination dynamics in the US: coverage velocity and carrying capacity based on socio-demographic vulnerability indices in California. J Immigr Minor Health. (2022) 24:18–30. 10.1007/s10903-021-01308-2 34797451 PMC8603654

[2] CDPH. Updated COVID-19 Vaccine Eligibility Guidelines. (2021). Available online at: https://www.cdph.ca.gov/Programs/CID/DCDC/Pages/COVID-19/VaccineAllocationGuidelines.aspx (accessed November 28, 2022).

[3] CDPH. Joint Statement on Western States Support for Expanded Eligibility for Children 6 Months and Older. (2022). Available online at: https://www.cdph.ca.gov/Programs/OPA/Pages/NR22-100.aspx (accessed November 28, 2022).

[4] Officer of Governor Gavin Newsom. All Californians 16+ Now Eligible for COVID-19 Vaccines | California Governor. (2021). Available online at: https://www.gov.ca.gov/2021/04/15/all-californians-16-now-eligible-for-covid-19-vaccines/ (accessed November 28, 2022).

[5] FDA. FDA Authorizes Pfizer-BioNTech COVID-19 Vaccine for Emergency Use in Children 5 through 11 Years of Age. (2021). Available online at: https://www.fda.gov/news-events/press-announcements/fda-authorizes-pfizer-biontech-covid-19-vaccine-emergency-use-children-5-through-11-years-age (accessed November 28, 2022).

[6] CDPH. Joint Statement from California Health and Human Services Agency Secretary Dr. Mark Ghaly and California Department of Public Health Director and State Public Health Officer Dr. Tomás Aragón. (2021). Available online at: https://www.cdph.ca.gov/Programs/OPA/Pages/NR21-320.aspx (accessed November 28 2022).

[7] CDPH. Joint Statement on Western States Recommendation of Expanded Pfizer-BioNTech Booster Eligibility for 16- and 17-Year-Olds. (2021). Available online at: https://www.cdph.ca.gov/Programs/OPA/Pages/NR21-350.aspx (accessed November 28, 2022).

[8] CDPH. Joint Statement on Western States Recommendation of Expanded Pfizer-BioNTech Booster Eligibility for 12- to 15-Year Olds. (2022). Available online at: https://www.cdph.ca.gov/Programs/OPA/Pages/NR22-005.aspx (accessed November 28, 2022).

[9] FDA. Coronavirus (COVID-19) Update: FDA Shortens Interval for Booster Dose of Moderna COVID-19 Vaccine to Five Months. (2022). Available online at: https://www.fda.gov/news-events/press-announcements/coronavirus-covid-19-update-fda-shortens-interval-booster-dose-moderna-covid-19-vaccine-five-months (accessed November 28, 2022).

[10] FDA. Coronavirus (COVID-19) Update: FDA Authorizes Second Booster Dose of Two COVID-19 Vaccines for Older and Immunocompromised Individuals. (2022). Available online at: https://www.fda.gov/news-events/press-announcements/coronavirus-covid-19-update-fda-authorizes-second-booster-dose-two-covid-19-vaccines-older-and (accessed November 28, 2022).

[11] CDPH. Joint Statement on Western States Support for Boosters for Children Ages 5-11. (2022). Available online at: https://www.cdph.ca.gov/Programs/OPA/Pages/NR22-088.aspx (accessed November 28, 2022).

[12] FDA. Coronavirus (COVID-19) Update: FDA Authorizes Moderna and Pfizer-BioNTech COVID-19 Vaccines for Children Down to 6 Months of Age. (2022). Available online at: https://www.fda.gov/news-events/press-announcements/coronavirus-covid-19-update-fda-authorizes-moderna-and-pfizer-biontech-covid-19-vaccines-children (accessed November 28, 2022).

[13] Population Clock (n.d.). Available online at: https://www.census.gov/popclock/ (accessed November 28, 2022).

[14] U.S. Census Bureau QuickFacts: California; United States (n.d.). Available online at: https://www.census.gov/quickfacts/fact/table/CA,US/PST045221#qf-headnote-b (accessed November 28, 2022).

[15] Kim C Yee R Bhatkoti R Carranza D. COVID-19 vaccine provider access and vaccination coverage among children aged 5-11 Years - United States, November 2021-January 2022. MMWR Morbid Mortal Wkly Rep. (2022) 71:378–83. 10.15585/mmwr.mm7110a4 35271559 PMC8911999

[16] DeCuir J Meng L Pan Y Vogt T Chatham-Stevens K Meador S. COVID-19 vaccine provider availability and vaccination coverage among children aged 5–11 years—United States, November 1, 2021–April 25, 2022. MMWR Morbid Mortal Wkly Rep. (2022) 71:847–51. 10.15585/mmwr.mm7126a3 35771688

[17] Santibanez TA Zhou T Black CL Vogt TM Bhavini Murthy P Pineau V. Sociodemographic variation in early uptake of COVID-19 vaccine and parental intent and attitudes toward vaccination of children aged 6 months−4 years — United States, July 1–29, 2022. Morbid Mortal Wkly Rep. (2022) 71:1479–84. 10.15585/mmwr.mm7146a3 36395039 PMC9707357

[18] Valier MR Elam-Evans LD Mu Y Santibanez TA Yankey D Zhou T. Racial and ethnic differences in COVID-19 vaccination coverage among children and adolescents aged 5–17 years and parental intent to vaccinate their children — national immunization survey–child COVID module, United States, December 2020–September 2022. MMWR Morbid Mortal Wkly Rep. (2023) 72:1–8. 10.15585/mmwr.mm7201a1 36602930 PMC9815155

[19] Hughes MM Wang A Grossman MK Pun E Whiteman A Deng L. County-level COVID-19 vaccination coverage and social vulnerability — United States, December 14, 2020–March 1, 2021. MMWR Morbid Mortal Wkly Rep. (2021) 70:431–6. 10.15585/mmwr.mm7012e1 33764963 PMC7993557

[20] Wolkin A Collier S House JS Reif D Motsinger-Reif A Duca L. Comparison of national vulnerability indices used by the centers for disease control and prevention for the COVID-19 response. Public Health Rep. (2022) 137:803. 10.1177/00333549221090262 35514159 PMC9257512

[21] Barry V Dasgupta S Weller DL Kriss JL Cadwell BL Rose C. Patterns in COVID-19 vaccination coverage, by social vulnerability and urbanicity— United States, December 14, 2020–May 1, 2021. Morbid Mortal Wkly Rep. (2021) 70:818. 10.15585/mmwr.mm7022e1 34081685 PMC8174677

[22] Murthy NC Zell E Fast HE Murthy BP Meng L Saelee R. Disparities in first dose COVID-19 vaccination coverage among children 5-11 years of age, United States. Emerg Infect Dis. (2022) 28:986–9. 10.3201/eid2805.220166 35226801 PMC9045440

[23] He K Mack WJ Neely M Lewis L Anand V. Parental perspectives on immunizations: impact of the COVID-19 pandemic on childhood vaccine hesitancy. J Community Health. (2022) 47:39. 10.1007/s10900-021-01017-9 34297272 PMC8299444

[24] COVID-19 Vaccine Progress Dashboard Data - COVID-19 Vaccines Administered By Demographics By County - California Open Data (n.d.). Available online at: https://data.ca.gov/dataset/covid-19-vaccine-progress-dashboard-data/resource/766edceb-8d41-4086-9856-4fb2538cec5f?inner_span=True (accessed November 28, 2022).

[25] CDC/ATSDR, Social Vulnerability Index (SVI) (n.d.). Available online at: https://www.atsdr.cdc.gov/placeandhealth/svi/index.html (accessed November 28, 2022).

[26] Los Angeles County Department of Public Health. COVID-19 Vaccination - How to Get Vaccinated (n.d.). Available online at: http://publichealth.lacounty.gov/acd/ncorona2019/vaccine/hcwsignup/ (accessed March 30, 2023).

[27] CA.gov. FACT SHEET: Ending the Pandemic Through Equitable Vaccine Administration (n.d.). California. Available online at: https://www.gov.ca.gov/wp-content/uploads/2021/03/Equitable-Vaccine-Administration-Fact-Sheet.pdf

[28] Ruiz JB Bell RA. Parental COVID-19 vaccine hesitancy in the United States. Public Health Rep. (2022) 137:1162–9. 10.1177/00333549221114346 35915993 PMC9574308

[29] Lendon PJ Santibanez AT Singleton AJ Lee TJ. Confidence in COVID-19 Vaccination of Children aged 12–17 years old, by Sociodemographic Factors Adult Respondents' Vaccination Status and Intent—Household Pulse Survey, United States, August 18–September 13, 2021. CDC (2021). Available online at: https://www.cdc.gov/vaccines/imz-managers/coverage/covidvaxview/pubs-resources/confidence-covid19-vaccination-children.html (accessed November 28, 2022).

[30] Carson SL Casillas A Castellon-Lopez Y Mansfield LN Morris D Barron J. COVID-19 vaccine decision-making factors in racial and ethnic minority communities in Los Angeles, California. JAMA Network Open. (2021) 4:e2127582. 10.1001/jamanetworkopen.2021.27582 34591103 PMC8485164

[31] Nguyen KH Nguyen K Geddes M Allen JD Corlin L. Trends in adolescent COVID-19 vaccination receipt and parental intent to vaccinate their adolescent children, United States. (2022) 54:733–42. 10.1080/07853890.2022.2045034 35238263 PMC8903754

[32] NASHP. States Address School Vaccine Mandates and Mask Mandates - The National Academy for State Health Policy. (2022). Available online at: https://www.nashp.org/states-enact-policies-to-support-students-transition-back-to-school/ (accessed November 28, 2022).

[33] Hamel L Lopes L Kearney A Stokes M Kirzinger A Sparks G. KFF COVID-19 Vaccine Monitor: Winter 2021 Update On Parents' Views Of Vaccines For Kids. KFF (2021). Available online at: https://www.kff.org/coronavirus-covid-19/poll-finding/kff-covid-19-vaccine-monitor-winter-2021-update-on-parents-views-of-vaccines/ (accessed November 28, 2022).

[34] Ryerson AB Rice CE Hung M-C Patel SA Weeks JD Kriss JL. Disparities in COVID-19 vaccination status, intent, and perceived access for noninstitutionalized adults, by disability status — National Immunization Survey Adult COVID Module, United States, May 30–June 26, 2021. MMWR Morbid Mortal Wkly Rep. (2021) 70:1365–71. 10.15585/mmwr.mm7039a2 34591826 PMC8486390

[35] CDC. People with Disabilities |COVID-19| (n.d.). Available online at: https://www.cdc.gov/ncbddd/humandevelopment/covid-19/people-with-disabilities.html (accessed November 28, 2022).

[36] Khairat S Zou B Adler-Milstein J. Factors and reasons associated with low COVID-19 vaccine uptake among highly hesitant communities in the US. Am J Infect Control. (2022) 50:262–7. 10.1016/j.ajic.2021.12.013 34995722 PMC8730806

