# Supplementary figures and images for: COVID-19 vaccination dynamics in the US: coverage velocity and carrying capacity based on socio-demographic vulnerability indices in California's pediatric population
Source: Front Public Health. 2023 May 9;11:1148200. doi: 10.3389/fpubh.2023.1148200 (PMC10203576; doi:10.3389/fpubh.2023.1148200)

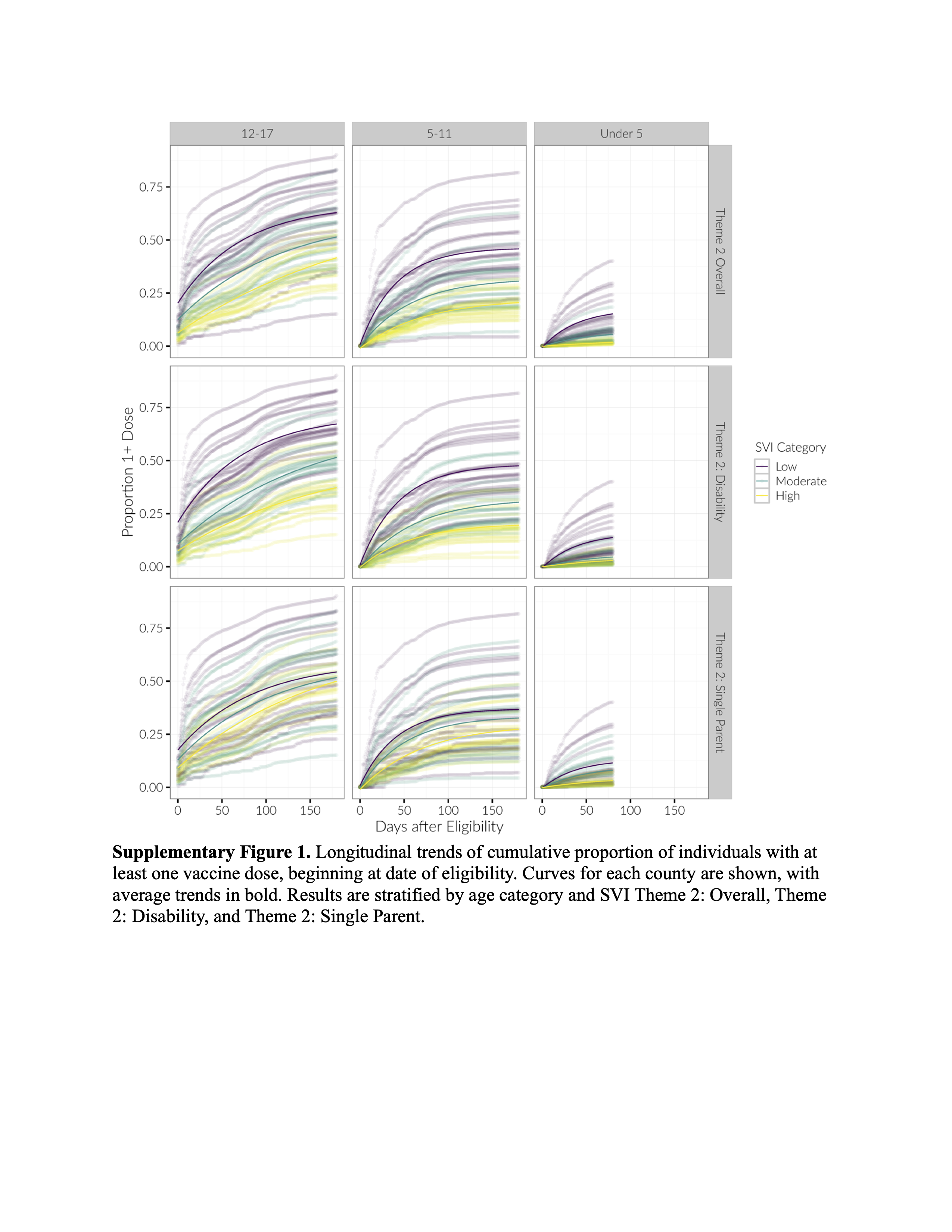

Supplement: Supplementary file 1 [file Image_1.PNG]

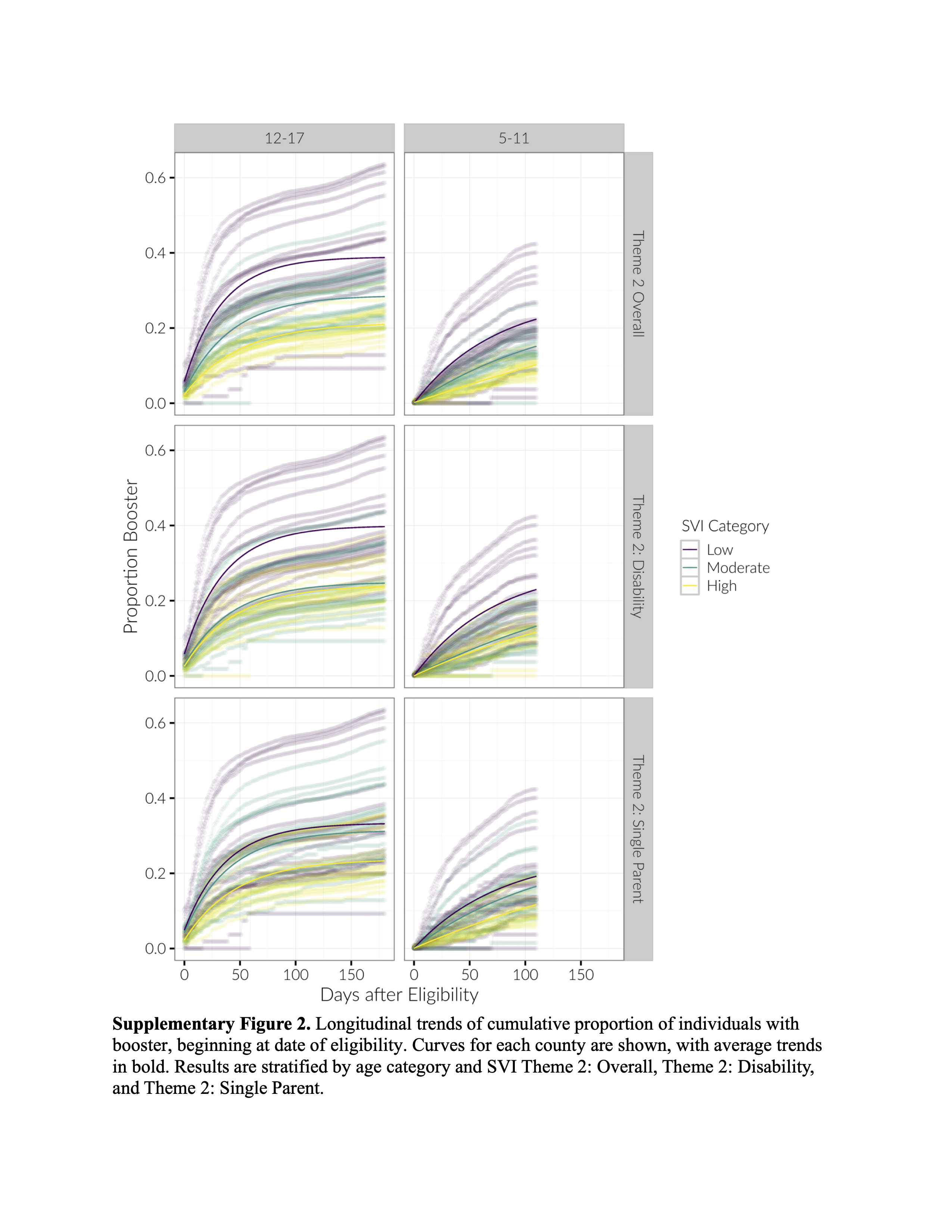

Supplement: Supplementary file 2 [file Image_2.PNG]

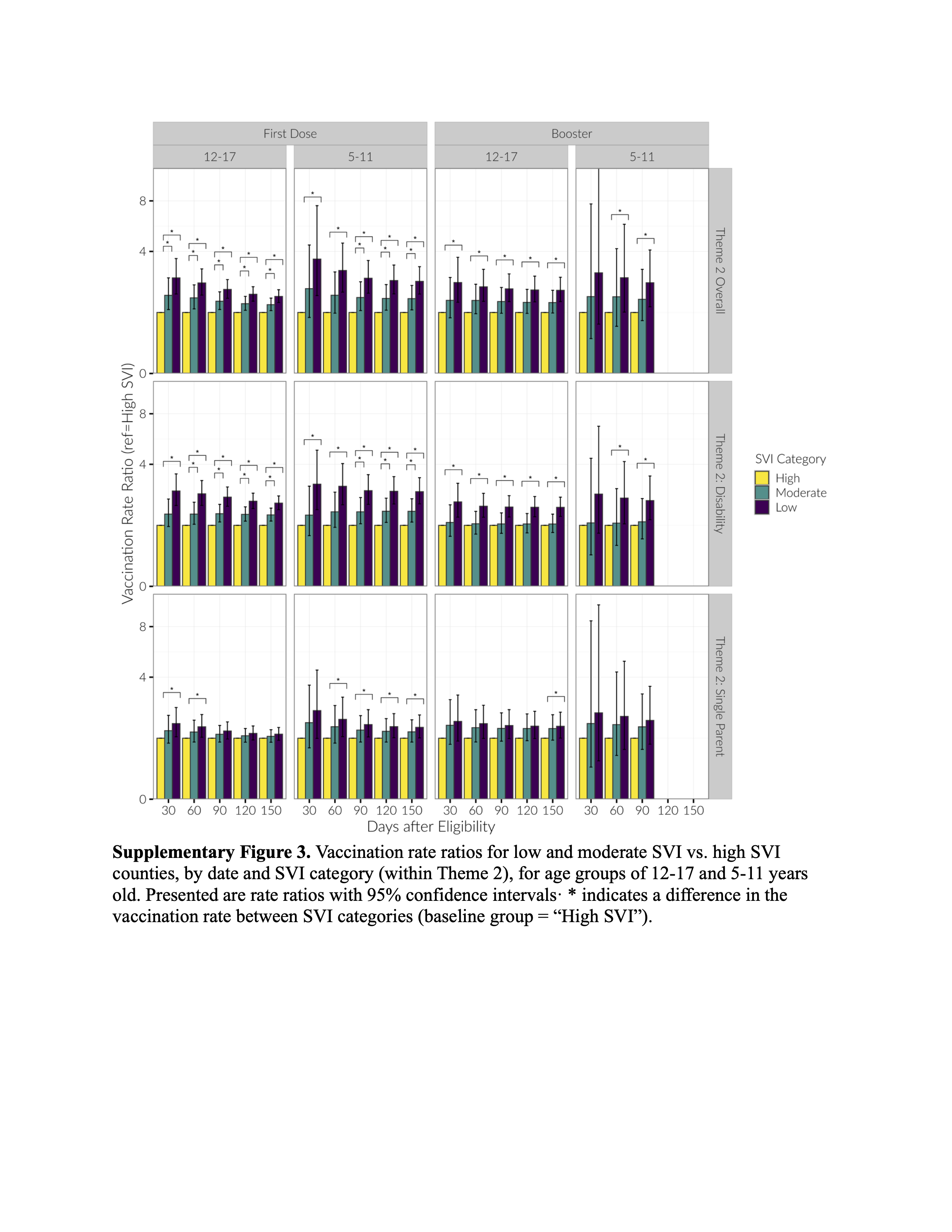

Supplement: Supplementary file 3 [file Image_3.PNG]
