# Supplementary material for: COVID-19 vaccination dynamics in the US: coverage velocity and carrying capacity based on socio-demographic vulnerability indices in California's pediatric population
Source: Front Public Health. 2023 May 9;11:1148200. doi: 10.3389/fpubh.2023.1148200 (PMC10203576; doi:10.3389/fpubh.2023.1148200)
Supplement: Supplementary file 4 [file Table_1.DOCX]

**Supplementary Table 1.** Snapshot Values by SVI theme and age group, from 30 days to 150 days after eligibility for first dose and booster dose. Note. svi = social vulnerability index; daycat = days after initial eligibility

| outcome | demographic_value | svi | daycat | Low / High | Moderate / High |
| --- | --- | --- | --- | --- | --- |
| First Dose | 12-17 | Theme 1 | 30 | 2.28 (1.58, 3.31)* | 1.44 (0.95, 2.18) |
| First Dose | 12-17 | Theme 1 | 60 | 2.02 (1.52, 2.7)* | 1.33 (0.97, 1.84) |
| First Dose | 12-17 | Theme 1 | 90 | 1.74 (1.39, 2.18)* | 1.22 (0.95, 1.57) |
| First Dose | 12-17 | Theme 1 | 120 | 1.57 (1.3, 1.89)* | 1.15 (0.94, 1.42) |
| First Dose | 12-17 | Theme 1 | 150 | 1.5 (1.26, 1.77)* | 1.13 (0.93, 1.36) |
| First Dose | 12-17 | Theme 2 | 30 | 2.45 (1.7, 3.55)* | 1.64 (1.1, 2.46)* |
| First Dose | 12-17 | Theme 2 | 60 | 2.2 (1.65, 2.94)* | 1.54 (1.12, 2.11)* |
| First Dose | 12-17 | Theme 2 | 90 | 1.9 (1.52, 2.37)* | 1.4 (1.1, 1.79)* |
| First Dose | 12-17 | Theme 2 | 120 | 1.69 (1.41, 2.02)* | 1.31 (1.07, 1.6)* |
| First Dose | 12-17 | Theme 2 | 150 | 1.6 (1.35, 1.89)* | 1.27 (1.06, 1.53)* |
| First Dose | 12-17 | Theme 3 | 30 | 0.68 (0.45, 1.03) | 1.23 (0.91, 1.65) |
| First Dose | 12-17 | Theme 3 | 60 | 0.68 (0.48, 0.95)* | 1.16 (0.91, 1.49) |
| First Dose | 12-17 | Theme 3 | 90 | 0.66 (0.5, 0.87)* | 1.11 (0.9, 1.35) |
| First Dose | 12-17 | Theme 3 | 120 | 0.68 (0.54, 0.86)* | 1.07 (0.9, 1.28) |
| First Dose | 12-17 | Theme 3 | 150 | 0.69 (0.56, 0.85)* | 1.06 (0.9, 1.25) |
| First Dose | 12-17 | Theme 4 | 30 | 0.93 (0.64, 1.36) | 1.02 (0.71, 1.47) |
| First Dose | 12-17 | Theme 4 | 60 | 0.93 (0.69, 1.27) | 1.01 (0.75, 1.36) |
| First Dose | 12-17 | Theme 4 | 90 | 0.93 (0.72, 1.19) | 0.99 (0.77, 1.26) |
| First Dose | 12-17 | Theme 4 | 120 | 0.92 (0.75, 1.15) | 0.97 (0.78, 1.2) |
| First Dose | 12-17 | Theme 4 | 150 | 0.93 (0.76, 1.14) | 0.96 (0.79, 1.18) |
| First Dose | 12-17 | Total | 30 | 1.82 (1.26, 2.63)* | 1.33 (0.88, 2) |
| First Dose | 12-17 | Total | 60 | 1.65 (1.23, 2.21)* | 1.24 (0.89, 1.72) |
| First Dose | 12-17 | Total | 90 | 1.45 (1.15, 1.83)* | 1.13 (0.88, 1.47) |
| First Dose | 12-17 | Total | 120 | 1.33 (1.09, 1.61)* | 1.07 (0.86, 1.33) |
| First Dose | 12-17 | Total | 150 | 1.28 (1.07, 1.53)* | 1.05 (0.86, 1.28) |
| First Dose | 5-11 | Theme 1 | 30 | 3.24 (1.54, 6.84)* | 1.72 (0.75, 3.92) |
| First Dose | 5-11 | Theme 1 | 60 | 2.69 (1.67, 4.31)* | 1.54 (0.91, 2.62) |
| First Dose | 5-11 | Theme 1 | 90 | 2.23 (1.59, 3.12)* | 1.39 (0.96, 2.03) |
| First Dose | 5-11 | Theme 1 | 120 | 2.11 (1.56, 2.86)* | 1.36 (0.97, 1.91) |
| First Dose | 5-11 | Theme 1 | 150 | 2.06 (1.54, 2.75)* | 1.34 (0.97, 1.85) |
| First Dose | 5-11 | Theme 2 | 30 | 3.51 (1.63, 7.57)* | 1.93 (0.84, 4.44) |
| First Dose | 5-11 | Theme 2 | 60 | 2.85 (1.78, 4.57)* | 1.64 (0.98, 2.77) |
| First Dose | 5-11 | Theme 2 | 90 | 2.44 (1.73, 3.43)* | 1.55 (1.06, 2.26)* |
| First Dose | 5-11 | Theme 2 | 120 | 2.32 (1.7, 3.16)* | 1.51 (1.08, 2.12)* |
| First Dose | 5-11 | Theme 2 | 150 | 2.28 (1.7, 3.06)* | 1.5 (1.09, 2.08)* |
| First Dose | 5-11 | Theme 3 | 30 | 0.76 (0.37, 1.53) | 1.4 (0.83, 2.36) |
| First Dose | 5-11 | Theme 3 | 60 | 0.71 (0.43, 1.16) | 1.28 (0.89, 1.84) |
| First Dose | 5-11 | Theme 3 | 90 | 0.7 (0.48, 1.02) | 1.21 (0.91, 1.6) |
| First Dose | 5-11 | Theme 3 | 120 | 0.68 (0.48, 0.97)* | 1.19 (0.92, 1.54) |
| First Dose | 5-11 | Theme 3 | 150 | 0.68 (0.48, 0.95)* | 1.18 (0.92, 1.51) |
| First Dose | 5-11 | Theme 4 | 30 | 0.91 (0.48, 1.73) | 1.01 (0.55, 1.85) |
| First Dose | 5-11 | Theme 4 | 60 | 0.92 (0.59, 1.44) | 0.98 (0.64, 1.52) |
| First Dose | 5-11 | Theme 4 | 90 | 0.92 (0.65, 1.3) | 0.98 (0.7, 1.38) |
| First Dose | 5-11 | Theme 4 | 120 | 0.91 (0.66, 1.26) | 0.97 (0.71, 1.33) |
| First Dose | 5-11 | Theme 4 | 150 | 0.91 (0.67, 1.24) | 0.97 (0.72, 1.31) |
| First Dose | 5-11 | Total | 30 | 2.5 (1.2, 5.21)* | 1.6 (0.71, 3.56) |
| First Dose | 5-11 | Total | 60 | 2.07 (1.29, 3.32)* | 1.42 (0.84, 2.38) |
| First Dose | 5-11 | Total | 90 | 1.78 (1.26, 2.53)* | 1.33 (0.91, 1.95) |
| First Dose | 5-11 | Total | 120 | 1.69 (1.23, 2.32)* | 1.29 (0.92, 1.83) |
| First Dose | 5-11 | Total | 150 | 1.65 (1.22, 2.24)* | 1.28 (0.92, 1.78) |
| Booster | 12-17 | Theme 1 | 30 | 2.27 (1.32, 3.89)* | 1.66 (0.93, 2.95) |
| Booster | 12-17 | Theme 1 | 60 | 1.92 (1.32, 2.81)* | 1.44 (0.96, 2.17) |
| Booster | 12-17 | Theme 1 | 90 | 1.83 (1.3, 2.56)* | 1.41 (0.98, 2.03) |
| Booster | 12-17 | Theme 1 | 120 | 1.79 (1.3, 2.47)* | 1.4 (0.99, 1.97) |
| Booster | 12-17 | Theme 1 | 150 | 1.79 (1.32, 2.43)* | 1.39 (1, 1.93)* |
| Booster | 12-17 | Theme 2 | 30 | 2.22 (1.36, 3.63)* | 1.44 (0.83, 2.48) |
| Booster | 12-17 | Theme 2 | 60 | 2.02 (1.4, 2.91)* | 1.43 (0.96, 2.14) |
| Booster | 12-17 | Theme 2 | 90 | 1.92 (1.39, 2.66)* | 1.39 (0.97, 1.99) |
| Booster | 12-17 | Theme 2 | 120 | 1.87 (1.38, 2.54)* | 1.36 (0.97, 1.9) |
| Booster | 12-17 | Theme 2 | 150 | 1.86 (1.39, 2.49)* | 1.35 (0.98, 1.86) |
| Booster | 12-17 | Theme 3 | 30 | 0.85 (0.5, 1.46) | 1.22 (0.78, 1.92) |
| Booster | 12-17 | Theme 3 | 60 | 0.9 (0.6, 1.34) | 1.2 (0.85, 1.7) |
| Booster | 12-17 | Theme 3 | 90 | 0.89 (0.61, 1.28) | 1.18 (0.86, 1.62) |
| Booster | 12-17 | Theme 3 | 120 | 0.88 (0.62, 1.25) | 1.18 (0.87, 1.59) |
| Booster | 12-17 | Theme 3 | 150 | 0.88 (0.63, 1.22) | 1.17 (0.88, 1.57) |
| Booster | 12-17 | Theme 4 | 30 | 0.9 (0.53, 1.54) | 0.99 (0.6, 1.65) |
| Booster | 12-17 | Theme 4 | 60 | 0.92 (0.61, 1.4) | 1.03 (0.7, 1.52) |
| Booster | 12-17 | Theme 4 | 90 | 0.92 (0.63, 1.33) | 1.01 (0.71, 1.45) |
| Booster | 12-17 | Theme 4 | 120 | 0.92 (0.64, 1.31) | 1.01 (0.72, 1.41) |
| Booster | 12-17 | Theme 4 | 150 | 0.92 (0.65, 1.29) | 1 (0.72, 1.38) |
| Booster | 12-17 | Total | 30 | 1.89 (1.1, 3.25)* | 1.41 (0.78, 2.53) |
| Booster | 12-17 | Total | 60 | 1.73 (1.14, 2.61)* | 1.4 (0.9, 2.17) |
| Booster | 12-17 | Total | 90 | 1.64 (1.14, 2.36)* | 1.36 (0.92, 2) |
| Booster | 12-17 | Total | 120 | 1.6 (1.13, 2.26)* | 1.34 (0.93, 1.93) |
| Booster | 12-17 | Total | 150 | 1.6 (1.15, 2.22)* | 1.33 (0.94, 1.88) |
| Booster | 5-11 | Theme 1 | 30 | 2.78 (0.61, 12.74) | 1.66 (0.31, 8.84) |
| Booster | 5-11 | Theme 1 | 60 | 2.47 (0.96, 6.35) | 1.67 (0.6, 4.64) |
| Booster | 5-11 | Theme 1 | 90 | 2.15 (1.13, 4.07)* | 1.49 (0.74, 2.99) |
| Booster | 5-11 | Theme 2 | 30 | 2.72 (0.65, 11.32) | 1.59 (0.33, 7.72) |
| Booster | 5-11 | Theme 2 | 60 | 2.46 (1.01, 5.98)* | 1.58 (0.6, 4.19) |
| Booster | 5-11 | Theme 2 | 90 | 2.21 (1.2, 4.1)* | 1.48 (0.75, 2.91) |
| Booster | 5-11 | Theme 3 | 30 | 0.75 (0.18, 3.11) | 1.13 (0.36, 3.53) |
| Booster | 5-11 | Theme 3 | 60 | 0.8 (0.32, 1.97) | 1.14 (0.54, 2.42) |
| Booster | 5-11 | Theme 3 | 90 | 0.81 (0.42, 1.58) | 1.15 (0.66, 2) |
| Booster | 5-11 | Theme 4 | 30 | 0.89 (0.21, 3.73) | 1.05 (0.28, 3.92) |
| Booster | 5-11 | Theme 4 | 60 | 0.93 (0.37, 2.31) | 1.03 (0.43, 2.44) |
| Booster | 5-11 | Theme 4 | 90 | 0.94 (0.49, 1.82) | 0.98 (0.52, 1.87) |
| Booster | 5-11 | Total | 30 | 2.38 (0.5, 11.35) | 1.53 (0.27, 8.55) |
| Booster | 5-11 | Total | 60 | 2.19 (0.82, 5.87) | 1.53 (0.53, 4.48) |
| Booster | 5-11 | Total | 90 | 1.91 (0.97, 3.77) | 1.41 (0.68, 2.96) |
