# Supplementary material for: COVID-19 vaccination dynamics in the US: coverage velocity and carrying capacity based on socio-demographic vulnerability indices in California's pediatric population
Source: Front Public Health. 2023 May 9;11:1148200. doi: 10.3389/fpubh.2023.1148200 (PMC10203576; doi:10.3389/fpubh.2023.1148200)
Supplement: Supplementary file 5 [file Table_2.DOCX]

**Supplementary Table 2.** Parameter estimates of n, r, and K for all age groups within Theme 2 (overall), Theme 2: Disability, and Theme 2: Single Parent. Note: SVI = social vulnerability index; r = growth parameter (coverage velocity); K = carrying capacity (estimated maximum proportion of individuals vaccinated)

| demographic_value | parm | svi | Theme 2 | Theme 2: Disability | Theme 2: Single Parent |
| --- | --- | --- | --- | --- | --- |
| 12-17 | n | Low | 0.203 (0.186, 0.219) | 0.209 (0.196, 0.223) | 0.176 (0.156, 0.195) |
| 12-17 | n | Moderate | 0.125 (0.109, 0.14) | 0.107 (0.094, 0.119) | 0.129 (0.111, 0.147) |
| 12-17 | n | High | 0.062 (0.048, 0.076) | 0.075 (0.063, 0.087) | 0.091 (0.073, 0.109) |
| 12-17 | r | Low | 0.014 (0.012, 0.016) | 0.013 (0.012, 0.015) | 0.012 (0.009, 0.014) |
| 12-17 | r | Moderate | 0.009 (0.007, 0.01) | 0.007 (0.005, 0.008) | 0.009 (0.007, 0.012) |
| 12-17 | r | High | 0.004 (0.003, 0.006) | 0.006 (0.004, 0.008) | 0.006 (0.004, 0.008) |
| 12-17 | K | Low | 0.667 (0.646, 0.688) | 0.718 (0.699, 0.737) | 0.594 (0.558, 0.63) |
| 12-17 | K | Moderate | 0.619 (0.568, 0.671) | 0.687 (0.617, 0.757) | 0.603 (0.553, 0.653) |
| 12-17 | K | High | 0.702 (0.525, 0.879) | 0.536 (0.443, 0.629) | 0.699 (0.581, 0.817) |
| 5-11 | r | Low | 0.025 (0.023, 0.026) | 0.023 (0.021, 0.024) | 0.024 (0.021, 0.027) |
| 5-11 | r | Moderate | 0.017 (0.015, 0.02) | 0.016 (0.014, 0.019) | 0.02 (0.017, 0.023) |
| 5-11 | r | High | 0.014 (0.011, 0.017) | 0.017 (0.014, 0.021) | 0.014 (0.011, 0.017) |
| 5-11 | K | Low | 0.464 (0.456, 0.472) | 0.484 (0.475, 0.492) | 0.371 (0.361, 0.382) |
| 5-11 | K | Moderate | 0.32 (0.307, 0.333) | 0.321 (0.307, 0.335) | 0.336 (0.323, 0.349) |
| 5-11 | K | High | 0.225 (0.206, 0.243) | 0.205 (0.193, 0.217) | 0.299 (0.275, 0.322) |
| Under 5 | r | Low | 0.025 (0.02, 0.031) | 0.025 (0.019, 0.032) | 0.026 (0.016, 0.035) |
| Under 5 | r | Moderate | 0.019 (0.005, 0.034) | 0.018 (-0.001, 0.037) | 0.021 (0.009, 0.033) |
| Under 5 | r | High | 0.008 (-0.033, 0.049) | 0.014 (-0.017, 0.045) | 0.014 (-0.015, 0.044) |
| Under 5 | K | Low | 0.176 (0.159, 0.193) | 0.159 (0.142, 0.177) | 0.132 (0.111, 0.153) |
| Under 5 | K | Moderate | 0.071 (0.043, 0.099) | 0.06 (0.026, 0.094) | 0.099 (0.072, 0.127) |
| Under 5 | K | High | 0.039 (-0.111, 0.189) | 0.044 (-0.018, 0.106) | 0.047 (-0.013, 0.108) |
