# Supplementary material for: COVID-19 vaccination dynamics in the US: coverage velocity and carrying capacity based on socio-demographic vulnerability indices in California's pediatric population
Source: Front Public Health. 2023 May 9;11:1148200. doi: 10.3389/fpubh.2023.1148200 (PMC10203576; doi:10.3389/fpubh.2023.1148200)
Supplement: Supplementary file 6 [file Table_3.DOCX]

**Supplementary Table 3.** Parameter estimates of n, r, and K for all age groups within Theme 2 (overall), Theme 2: Disability, and Theme 2: Single Parent for booster shot. Note: SVI = social vulnerability index; r = growth parameter (coverage velocity); k = carrying capacity (estimated maximum proportion of individuals vaccinated)

| demographic_value | parm | svi | Theme 2 | Theme 2: Disability | Theme 2: Single Parent |
| --- | --- | --- | --- | --- | --- |
| 12-17 | n | Low | 0.058 (0.045, 0.071) | 0.058 (0.045, 0.071) | 0.048 (0.033, 0.064) |
| 12-17 | n | Moderate | 0.031 (0.019, 0.044) | 0.03 (0.017, 0.043) | 0.042 (0.027, 0.057) |
| 12-17 | n | High | 0.023 (0.011, 0.034) | 0.026 (0.014, 0.038) | 0.023 (0.008, 0.037) |
| 12-17 | r | Low | 0.029 (0.027, 0.032) | 0.028 (0.026, 0.03) | 0.027 (0.024, 0.03) |
| 12-17 | r | Moderate | 0.024 (0.021, 0.026) | 0.024 (0.021, 0.027) | 0.025 (0.022, 0.028) |
| 12-17 | r | High | 0.02 (0.017, 0.023) | 0.022 (0.019, 0.025) | 0.022 (0.018, 0.025) |
| 12-17 | K | Low | 0.389 (0.384, 0.395) | 0.399 (0.394, 0.405) | 0.334 (0.327, 0.341) |
| 12-17 | K | Moderate | 0.287 (0.281, 0.294) | 0.25 (0.243, 0.256) | 0.315 (0.307, 0.322) |
| 12-17 | K | High | 0.215 (0.207, 0.223) | 0.242 (0.235, 0.249) | 0.238 (0.229, 0.247) |
| 5-11 | r | Low | 0.014 (0.011, 0.016) | 0.014 (0.011, 0.016) | 0.013 (0.009, 0.016) |
| 5-11 | r | Moderate | 0.007 (0.004, 0.011) | 0.004 (0, 0.009) | 0.008 (0.004, 0.012) |
| 5-11 | r | High | 0.002 (-0.004, 0.007) | 0.005 (0.001, 0.01) | 0.004 (-0.002, 0.009) |
| 5-11 | K | Low | 0.289 (0.259, 0.318) | 0.296 (0.267, 0.325) | 0.255 (0.218, 0.292) |
| 5-11 | K | Moderate | 0.272 (0.179, 0.364) | 0.348 (0.069, 0.627) | 0.276 (0.193, 0.359) |
| 5-11 | K | High | 0.66 (-1.583, 2.903) | 0.259 (0.089, 0.429) | 0.361 (-0.112, 0.833) |
