# Supplementary material for: COVID-19 vaccination dynamics in the US: coverage velocity and carrying capacity based on socio-demographic vulnerability indices in California's pediatric population
Source: Front Public Health. 2023 May 9;11:1148200. doi: 10.3389/fpubh.2023.1148200 (PMC10203576; doi:10.3389/fpubh.2023.1148200)
Supplement: Supplementary file 7 [file Table_4.DOCX]

**Supplementary Table 4.** Snapshot Values by SVI theme and age group, from 30 days to 150 days after eligibility for first dose and booster dose within Theme 2: Overall, Theme 2: Disability, and Theme 2: Single Parent. Note. svi = social vulnerability index; daycat = days after initial eligibility

| outcome | demographic_value | svi | daycat | Low / High | Moderate / High |
| --- | --- | --- | --- | --- | --- |
| First Dose | 12-17 | Theme 2 Overall | 30 | 2.45 (1.7, 3.55)* | 1.64 (1.1, 2.46)* |
| First Dose | 12-17 | Theme 2 Overall | 60 | 2.2 (1.65, 2.94)* | 1.54 (1.12, 2.11)* |
| First Dose | 12-17 | Theme 2 Overall | 90 | 1.9 (1.52, 2.37)* | 1.4 (1.1, 1.79)* |
| First Dose | 12-17 | Theme 2 Overall | 120 | 1.69 (1.41, 2.02)* | 1.31 (1.07, 1.6)* |
| First Dose | 12-17 | Theme 2 Overall | 150 | 1.6 (1.35, 1.89)* | 1.27 (1.06, 1.53)* |
| First Dose | 12-17 | Theme 2: Disability | 30 | 2.44 (1.76, 3.4)* | 1.4 (0.96, 2.05) |
| First Dose | 12-17 | Theme 2: Disability | 60 | 2.3 (1.76, 3.01)* | 1.4 (1.03, 1.9)* |
| First Dose | 12-17 | Theme 2: Disability | 90 | 2.14 (1.72, 2.65)* | 1.42 (1.11, 1.8)* |
| First Dose | 12-17 | Theme 2: Disability | 120 | 1.95 (1.63, 2.33)* | 1.39 (1.14, 1.7)* |
| First Dose | 12-17 | Theme 2: Disability | 150 | 1.86 (1.58, 2.19)* | 1.37 (1.14, 1.64)* |
| First Dose | 12-17 | Theme 2: Single Parent | 30 | 1.54 (1.05, 2.26)* | 1.26 (0.84, 1.89) |
| First Dose | 12-17 | Theme 2: Single Parent | 60 | 1.41 (1.03, 1.92)* | 1.21 (0.88, 1.68) |
| First Dose | 12-17 | Theme 2: Single Parent | 90 | 1.25 (0.97, 1.61) | 1.13 (0.88, 1.46) |
| First Dose | 12-17 | Theme 2: Single Parent | 120 | 1.16 (0.94, 1.44) | 1.08 (0.87, 1.34) |
| First Dose | 12-17 | Theme 2: Single Parent | 150 | 1.14 (0.93, 1.38) | 1.06 (0.87, 1.3) |
| First Dose | 5-11 | Theme 2 Overall | 30 | 3.51 (1.63, 7.57)* | 1.93 (0.84, 4.44) |
| First Dose | 5-11 | Theme 2 Overall | 60 | 2.85 (1.78, 4.57)* | 1.64 (0.98, 2.77) |
| First Dose | 5-11 | Theme 2 Overall | 90 | 2.44 (1.73, 3.43)* | 1.55 (1.06, 2.26)* |
| First Dose | 5-11 | Theme 2 Overall | 120 | 2.32 (1.7, 3.16)* | 1.51 (1.08, 2.12)* |
| First Dose | 5-11 | Theme 2 Overall | 150 | 2.28 (1.7, 3.06)* | 1.5 (1.09, 2.08)* |
| First Dose | 5-11 | Theme 2: Disability | 30 | 2.81 (1.58, 4.98)* | 1.36 (0.69, 2.7) |
| First Dose | 5-11 | Theme 2: Disability | 60 | 2.69 (1.79, 4.05)* | 1.49 (0.93, 2.38) |
| First Dose | 5-11 | Theme 2: Disability | 90 | 2.46 (1.8, 3.35)* | 1.49 (1.05, 2.12)* |
| First Dose | 5-11 | Theme 2: Disability | 120 | 2.43 (1.82, 3.23)* | 1.51 (1.1, 2.09)* |
| First Dose | 5-11 | Theme 2: Disability | 150 | 2.41 (1.83, 3.17)* | 1.51 (1.11, 2.06)* |
| First Dose | 5-11 | Theme 2: Single Parent | 30 | 2.11 (0.99, 4.48) | 1.57 (0.71, 3.49) |
| First Dose | 5-11 | Theme 2: Single Parent | 60 | 1.71 (1.05, 2.8)* | 1.41 (0.85, 2.36) |
| First Dose | 5-11 | Theme 2: Single Parent | 90 | 1.5 (1.04, 2.15)* | 1.28 (0.88, 1.88) |
| First Dose | 5-11 | Theme 2: Single Parent | 120 | 1.41 (1.02, 1.97)* | 1.24 (0.88, 1.74) |
| First Dose | 5-11 | Theme 2: Single Parent | 150 | 1.39 (1.01, 1.91)* | 1.22 (0.88, 1.69) |
| Booster | 12-17 | Theme 2 Overall | 30 | 2.22 (1.36, 3.63)* | 1.44 (0.83, 2.48) |
| Booster | 12-17 | Theme 2 Overall | 60 | 2.02 (1.4, 2.91)* | 1.43 (0.96, 2.14) |
| Booster | 12-17 | Theme 2 Overall | 90 | 1.92 (1.39, 2.66)* | 1.39 (0.97, 1.99) |
| Booster | 12-17 | Theme 2 Overall | 120 | 1.87 (1.38, 2.54)* | 1.36 (0.97, 1.9) |
| Booster | 12-17 | Theme 2 Overall | 150 | 1.86 (1.39, 2.49)* | 1.35 (0.98, 1.86) |
| Booster | 12-17 | Theme 2: Disability | 30 | 1.92 (1.29, 2.85)* | 1.1 (0.67, 1.78) |
| Booster | 12-17 | Theme 2: Disability | 60 | 1.73 (1.28, 2.32)* | 1.05 (0.73, 1.51) |
| Booster | 12-17 | Theme 2: Disability | 90 | 1.69 (1.29, 2.21)* | 1.05 (0.76, 1.45) |
| Booster | 12-17 | Theme 2: Disability | 120 | 1.68 (1.3, 2.17)* | 1.05 (0.77, 1.42) |
| Booster | 12-17 | Theme 2: Disability | 150 | 1.68 (1.31, 2.14)* | 1.04 (0.78, 1.4) |
| Booster | 12-17 | Theme 2: Single Parent | 30 | 1.63 (0.91, 2.91) | 1.47 (0.81, 2.66) |
| Booster | 12-17 | Theme 2: Single Parent | 60 | 1.53 (0.99, 2.37) | 1.38 (0.88, 2.15) |
| Booster | 12-17 | Theme 2: Single Parent | 90 | 1.46 (0.99, 2.16) | 1.35 (0.9, 2) |
| Booster | 12-17 | Theme 2: Single Parent | 120 | 1.43 (0.99, 2.08) | 1.34 (0.92, 1.95) |
| Booster | 12-17 | Theme 2: Single Parent | 150 | 1.43 (1, 2.03)* | 1.34 (0.94, 1.92) |
| Booster | 5-11 | Theme 2 Overall | 30 | 2.72 (0.65, 11.32) | 1.59 (0.33, 7.72) |
| Booster | 5-11 | Theme 2 Overall | 60 | 2.46 (1.01, 5.98)* | 1.58 (0.6, 4.19) |
| Booster | 5-11 | Theme 2 Overall | 90 | 2.21 (1.2, 4.1)* | 1.48 (0.75, 2.91) |
| Booster | 5-11 | Theme 2: Disability | 30 | 2.29 (0.76, 6.88) | 1.08 (0.27, 4.4) |
| Booster | 5-11 | Theme 2: Disability | 60 | 2.1 (1.05, 4.18)* | 1.07 (0.45, 2.56) |
| Booster | 5-11 | Theme 2: Disability | 90 | 1.98 (1.19, 3.27)* | 1.12 (0.61, 2.07) |
| Booster | 5-11 | Theme 2: Single Parent | 30 | 2 (0.4, 10.15) | 1.54 (0.28, 8.54) |
| Booster | 5-11 | Theme 2: Single Parent | 60 | 1.84 (0.67, 5.11) | 1.49 (0.51, 4.35) |
| Booster | 5-11 | Theme 2: Single Parent | 90 | 1.67 (0.81, 3.42) | 1.41 (0.67, 2.98) |
|  |  |  |  |  |  |
|  |  |  |  |  |  |
|  |  |  |  |  |  |
|  |  |  |  |  |  |
|  |  |  |  |  |  |
|  |  |  |  |  |  |
